# Proximity labeling in neuroscience: decoding molecular landscapes for precision neurology

**DOI:** 10.1186/s40035-026-00534-8

**Published:** 2026-01-29

**Authors:** Xia Gao, Jianjun Lu, Peipei Chen, Xinna Wang, Longlong Zheng, Yuyin Shao, Huali Shen, Qian Yang

**Affiliations:** 1 Department of Experimental Surgery, Tangdu Hospital, The Fourth Military Medical University, Xi’an, 710038 China; 2 Institutes of Biomedical Sciences, Fudan University, Shanghai, 200032 China; 3 Department of Interventional Radiology, Tangdu Hospital, The Fourth Military Medical University, Xi’an, 710038 China; 4 Leibniz-Forschungsinstitut für Molekulare Pharmakologie, 13125 Berlin, Germany

**Keywords:** Proximity labeling, Neurological disorders, Protein-protein interaction, Biomarker discovery, Therapeutic targets, Spatial proteomics

## Abstract

The intricate cellular architecture and dynamic molecular interplay in the nervous system have long challenged mechanistic studies of neurological diseases. Conventional approaches often miss the transient, low-affinity, or spatially confined interactions that underlie neural homeostasis and pathogenesis. Proximity labeling (PL) technologies overcome this limitation by enabling in situ capture of these elusive molecular events within living systems. Through spatially restricted biotinylation, PL methods, including engineered biotin ligases (e.g., TurboID), peroxidases (e.g., APEX2), and emerging photocatalytic platforms, allow high-resolution mapping of proteomes and interactomes within defined subcellular compartments, cell types, and cell-cell interfaces. In this review, we systematically outline the principles of PL and its transformative applications in constructing molecular atlases of the nervous system. We highlight how these tools are revolutionizing our understanding of brain function by elucidating pathophysiological mechanisms in Alzheimer’s disease, Parkinson’s disease and other neurological disorders. Furthermore, we discuss how PL accelerates the translation of basic research into clinical practice by facilitating the discovery of mechanistic biomarkers and druggable targets. Finally, we address current challenges and future directions, including integration with multi-omics and single-cell methodologies, and conclude that PL can advance precision neurology by bridging molecular neurobiology with therapeutic innovation.

## Introduction

The mammalian nervous system executes complex behaviors through specialized, precisely positioned, and interacting cell types [1–3]. A sophisticated network of molecular interactions underpins the functions of neurons and other brain cell types. These interactions modulate signaling pathways, regulate neurotransmitter release, and sustain proper neural connectivity [4]. Disruptions in protein-protein interaction (PPI) network are associated with synaptic communication deficits, protein aggregation, and dysregulated cell death pathways, collectively contributing to the pathogenesis of various neurodegenerative diseases and neurodevelopmental disorders [5–7].

Spatial compartmentalization ensures molecular complexes and biological processes occur in precise subcellular localizations [8]. The functions of any biomolecule are determined not only by its intrinsic properties but also by its spatial context within the cell [9]. Consequently, systems-level mapping of spatial interaction networks is required to obtain mechanistic understanding of cellular processes [10]. Although traditional methods such as co-immunoprecipitation (Co-IP) and subcellular fractionation can provide some information [11, 12], they have inherent limitations in preserving the spatial context of these interactions and in capturing transient or low-affinity interactions.

Recently, proximity labeling (PL), a cornerstone of “spatiomics”, has emerged as a powerful approach to mapping protein proximity networks and interactomes in living systems [13–16]. Newer platforms such as TurboID and ascorbate peroxidase (APEX2), building upon early biotin ligase-based strategies that have characterized synaptic proteomes in living cells and mice [17, 18], enable high-resolution “molecular spatiomics” by capturing PPIs under near-physiological conditions while preserving native membrane integrity and protein complexes [19]. Critically, PL enables mapping of otherwise unpurifiable subcellular regions within their native context (e.g., the synaptic cleft [17]) without physical isolation. This avoids detergent-induced artifacts and improves recovery of fragile membrane proteins, including receptors, ion channels, and transporters. PL has been applied to delineate subcellular-compartment proteomes, cell-type-specific spatial proteomes, cell-surface proteomes, and cell-cell interaction networks [20–23]. Moreover, PL is evolving from a proteomic tool into a dynamic “molecular microscope” for neural-circuit dissection, with potentials to redefine diagnosis and therapy through discovery of mechanistic biomarkers and druggable targets.

## PL techniques

### Overview of PL

**Fig. 1:**
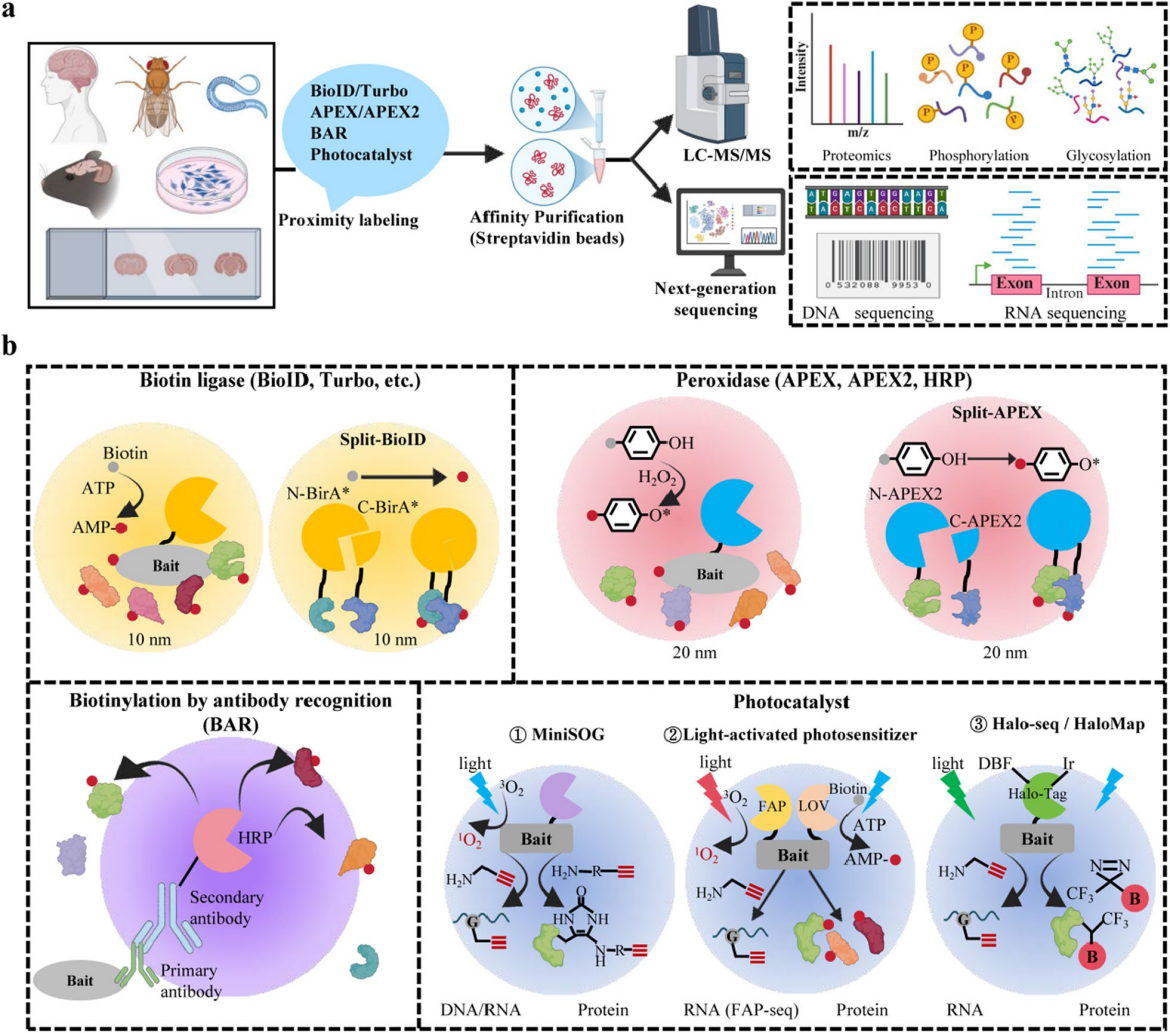
Overview of proximity labeling (PL) strategies and workflow. **a** General PL workflow. PL is performed in diverse experimental systems (in vitro, in vivo, or ex vivo), followed by enrichment of biotinylated molecules and analysis via mass spectrometry or next-generation sequencing. **b** Classification and schematic representation of major PL techniques. A bait protein is fused to an engineered enzyme capable of catalyzing biotinylation. Upon addition of substrate (e.g., biotin or biotin-phenol), the enzyme labels proximal endogenous biomolecules. Created with BioRender.com

PL enables the mapping of molecular interactions and spatial neighborhoods in living cells and organisms. The core principle of PL involves fusing a protein of interest (POI) or a “bait” to an engineered enzyme that catalyzes the generation of reactive molecules (e.g., biotin-AMP or biotin-phenoxyl radicals) upon substrate addition. These reactive species covalently tag endogenous biomolecules within a short radius (typically ~10–20 nm) to the bait. The biotinylated molecules can then be affinity-purified and identified by mass spectrometry (MS) or next-generation sequencing, allowing for the reconstruction of local molecular landscapes under near-physiological conditions (Fig. 1a) [24–26].

### Classification of PL techniques

Current PL platforms are primarily categorized by the enzymes used, each with distinct mechanisms and trade-offs (Fig. 1b).

Biotin ligases, such as BioID, TurboID, miniTurbo, BASU (a mutant BirA* engineered from *Bacillus subtilis*), and AirID, use ATP to convert biotin into a reactive biotin-AMP intermediate that labels proximal lysine residues. TurboID and its derivatives are widely used due to their rapid kinetics (labeling within minutes). However, they can deplete endogenous biotin pools and exhibit basal activity [27–29].

Peroxidases, such as APEX/APEX2 and horseradish peroxidase (HRP), utilize H_2_O_2_ to generate highly reactive, short-lived biotin-phenoxyl radicals that label electron-rich amino acids (e.g., tyrosine, tryptophan, cysteine, histidine). They offer ultrafast labeling (~ 1 min) and high temporal resolution, making them ideal for capturing rapid biological processes. However, the required H_2_O_2_ can be cytotoxic and perturb the redox-sensitive pathways, limiting some in vivo applications [15, 30–32].

In Split-PL variants, PL enzyme is split into two inactive fragments, each fused to two different POI proteins or localized to distinct compartments. Proximity-driven reconstitution restores the enzymetic activity and restricts labeling to specific PPIs, compartment interfaces, or known complexes, improving the specificity for conditional or inducible PPIs.

Antibody-based PL, such as biotinylation by antibody recognition (BAR), enables the mapping of PPIs in fixed cells and clinically relevant human tissues without genetic modification [33]. This technique employs a primary antibody to target an endogenous protein, followed by an HRP-conjugated secondary antibody. Upon substrate addition, the HRP enzyme is activated, generating reactive species that can covalently tag nearby proteins [34]. This method allows stringent purification and highly efficient proteomic analysis of native interactomes under pathophysiological conditions, effectively bridging basic and clinical neuroscience. However, the method is critically dependent on antibody specificity and affinity, necessitating rigorous validation. In addition, it offers limited spatial resolution due to the labeling radius that can extend to hundreds of nanometers. Additionally, the requirement of sample fixation and the use of peroxide make it unsuitable for live-cell applications.

Photocatalytic PL (Photo-PL) utilizes diverse light-activated catalysts, including genetically encoded proteins (e.g., miniSOG [35], fluorescent activator protein [36], or light-oxygen-voltage domains [LOV] [21]), transition-metal complexes (e.g., iridium- or ruthenium-based catalysts [37–40]), and organic dyes (e.g., Eosin Y [41], rhodamine 123 [42], dibenzylfluorescein), to generate reactive species such as singlet oxygen [35, 43], quinone methides [36], carbenes [44, 45], or phenoxy radicals [46], for spatially controlled biomolecular labeling in living systems [47]. This approach achieves exceptional spatiotemporal precision and bioorthogonality, enabling high-resolution mapping of dynamic subcellular processes, as demonstrated in mitochondrial proteomics with iridium-based CAT-Prox [37], RNA localization studies using Halo-seq [39], stress granule (SG) dynamics with HaloMap [40], and T-cell immune-synapse surface networks via Eosin Y-mediated labeling [41]. However, the broader application of Photo-PL in vivo is constrained by limited light penetration in tissues, challenges in probe delivery, and the need to optimize reaction kinetics under physiological conditions.

### Applications of PL techniques

**Fig. 2:**
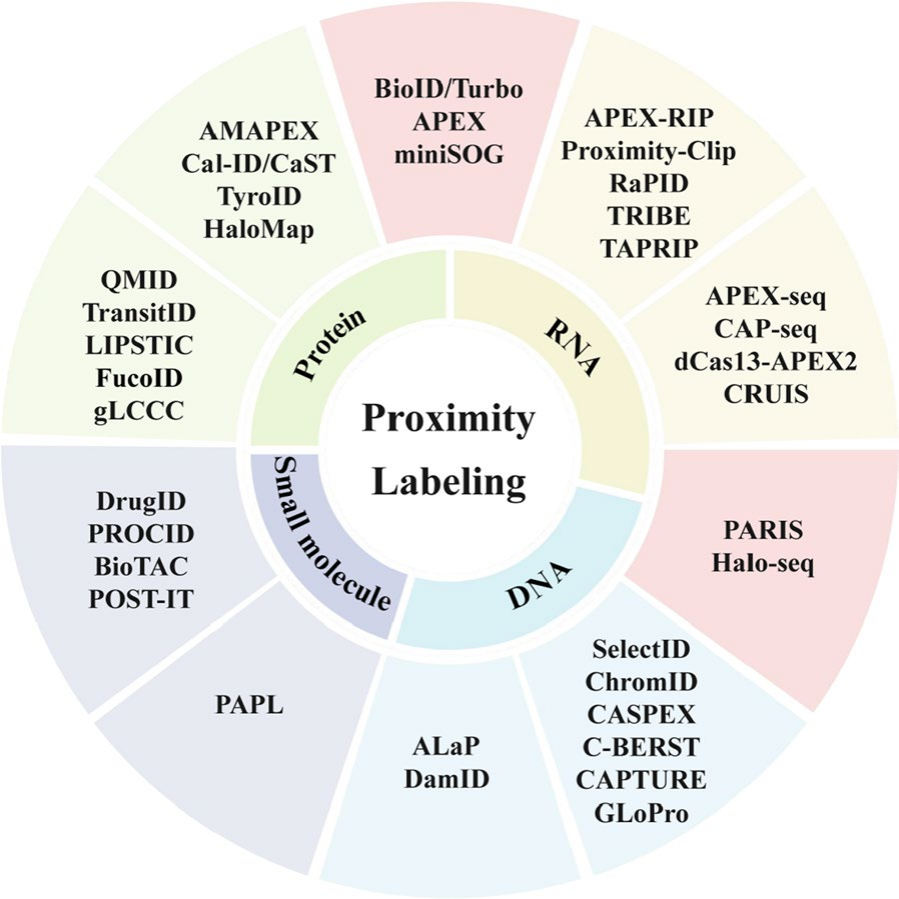
Applications of PL techniques for spatial interactome mapping. PL methods are categorized by target molecule class: Proteins (e.g., AMAPEX, Cal-ID/CaST, TyroID, HaloMap for identifying protein-protein interactions; QMID, TransitID, LIPSTIC, FucoID, gLCCC for mapping cell-cell interactions), RNA (e.g., APEX-RIP, Proximity-Clip, RaPID, TRIBE, TAPRIP for identifying RNAs interacting with a POI; APEX-seq, CAP-seq, dCas13-APEX2, CRUIS for mapping RNA-binding proteins), DNA (e.g., ALaP and DamID for mapping chromatin interactions; SelectID, ChromID, CASPEX, C-BERST, CAPTURE, GLoPro for identifying proteins associated with specific DNA loci), and Small molecules (e.g., PAPL for identifying cellular organelle lipids; DrugID, PROCID, BioTAC, POST-IT for capturing small molecules and their interacting partners). The red modules highlight multi-class detection: protein/RNA (BioID/Turbo, APEX, miniSOG) and RNA/DNA (PARIS, Halo-seq). Created with BioRender.com

PL has evolved into a versatile platform for interactome mapping across all major biomolecule classes—proteins, RNA, DNA, and small molecules, with spatial resolution [34, 48, 49] (Table 1). A critical factor in PL labeling of these biomolecule classes is the selectivity of the labeling chemistry. For example, PL labeling of proteins is often directed at specific reactive amino acids. In contrast, the labeling of nucleic acids is less specific. The generated radicals (e.g., from APEX) react promiscuously with the electron-rich nucleobases of proximal RNAs or DNAs. PL labeling of lipids presents a distinct challenge due to the hydrocarbon-dominated structure. A recent study demonstrated photocatalytic lipid labeling using a lipophilic azide probe. Upon blue light exposure, organelle-localized photocatalysts activate the probe to generate triplet nitrenes, which then selectively label nearby lipids with nucleophilic head-groups (e.g., phosphatidylethanolamine) via covalent bonding [50]. The primary advantage of these approaches is the ability to capture spatially resolved interactomes in live cells. However, there is a key limitation due to the inherent chemical bias; nucleic acid labeling lacks base specificity, and lipid labeling often under-represents saturated and neutral lipid classes that lack these reactive moieties. In the following sections, we will elaborate on these diverse applications (Fig. 2, Table 1**)**, underscoring the role of PL as a foundational technology for multidimensional molecular mapping.

**Table 1:** Comparison of key PL technologies for spatial molecular landscape mapping

Biomolecules	Method	Enzyme type/Principle	Advantages	Limitations	Applications	Ref.
Protein	Protein molecular profile					
	BioID/TurboID/BASU	Biotin ligase	Non-toxic for in vivo applications.	Poor temporal resolution as a result of low catalytic activity.	Identifying proximal proteins in living cells.	[16]
	APEX/APEX2/HPR	Peroxidase	High temporal resolution, versatility for both protein and RNA labeling.	Limited application in vivo because of the toxicity of H_2_O_2._	Mapping proteomes and interactomes rapidly (minutes) in living or fixed cells.	[16]
	BAR	Antibody-targeted, HRP-based technique	Enables mapping interactomes of specific endogenous proteins and PTM profiling in fixed cells and primary human tissues, without genetic manipulation.	Limited by a large labeling radius and a critical dependence on antibody quality, restricting its use to fixed-cell contexts.	Identifying the interactors of lamin A/C in immortalized cell lines, primary cell culture, and primary human muscle and adipose tissues.	[33]
	AMAPEX	Antibody-targeted, APEX2-based technique	Enables labeling of endogenous proteins and specific PTMs without genetic manipulation.	Limited by antibody quality and requires cell permeabilization.	Maps interactomes of specific endogenous protein states, like histone modifications.	[53] [54]
	TyroID	Utilizes recombinant tyrosinase to generate reactive o-quinone intermediates.	High spatiotemporal resolution and in vivo-compatible, minimal perturbation to native biological systems.	Limited to extracellular environments and tyrosine-rich regions, potentially missing proteins information.	Mapping dynamic extracellular proteomes in vivo, such as tumor microenvironments and brain region-specific surfaceomes.	[61]
	HaloMap	Photo-PL, a HaloTag-fused protein to recruit a photosensitizer molecule.	High spatiotemporal control for mapping dynamic processes in living cells.	Requires genetic engineering to express the HaloTag fusion and exogenous delivery of the photosensitizer ligand.	Unraveling intracellular protein interactomes and dynamics of membraneless organelles, like stress granules.	[40]
	MiniSOG	Photo-activated to generate singlet oxygen upon light, covalently labeling neighboring proteins or RNA/DNA.	Enables identification of weak, transient interactions in live cells with high spatiotemporal control.	Requires genetic manipulation and may cause cellular photo damage.	Mapping PPIs and subcellular molecular landscapes in live cells.	[36]
	Cal-ID/CaST	Biotin ligase, calcium-dependent.	Provides permanent, transcription-independent molecular recording of transient Ca^2+^ signals with high spatial resolution.	Requires minutes for detectable labeling to miss transient information, and could bias toward calmodulin-binding proteins.	Capturing calcium-induced biotinylation upon neuronal activation in primary cortical neurons and kainic-acid-induced seizures.	[62]
	Cell-cell interactions					
	QMID	Utilizes enzyme-generated QM electrophiles that diffuse micrometers to covalently label neighboring cells without direct cell-cell contact.	Enables recording of cell spatial organization at cellular-scale resolution (~μm), capturing both direct and indirect cell communications in native tissues.	Limited by the diffusion range and stability of QM electrophiles, potentially labeling non-specific cells beyond the intended proximity range.	Dissecting immune niches and tumor microenvironments by labeling and isolating spatially proximal cells for downstream single-cell analysis.	[67]
	TransitID	Utilizes two distinct PL enzymes targeted to different cellular compartments to sequentially biotinylate proteins that traffic between various locations.	Enables nanometer-resolution mapping of endogenous proteome trafficking dynamics between compartments and even between different cells in co-culture systems.	Requires careful optimization of sequential labeling timing and enzyme targeting, and may miss very rapid or transient trafficking events due to the time needed for biotinylation.	Mapping protein trafficking between organelles (e.g., cytosol-mitochondria) and intercellular communication between immune and cancer cells.	[69]
	LIPSTIC	Utilizes sortase A to catalyze transpeptidation between a labeled substrate and cell surface proteins upon receptor-ligand engagement.	Enables monitoring of dynamic cell-cell interactions in vivo with single-cell resolution and compatibility with downstream analysis.	Requires genetic engineering to express sortase-acceptable substrates and limited to extracellular interactions.	Quantifying transient intercellular communication events in immunology (e.g., T cell-DC interactions).	[68]
	FucoID	Expresses site-specific fucosyltransferase (sFT) in cells or uses antibody conjugates (Ab-sFT) to confirm cell-cellinteractions.	Enables revealing interactions of different molecular pairs, such as peptide-major histocompatibility complex-T cell receptor, chimeric antigen receptor-antigen, and PD-1–PD-L1.	Limited by its dependence on having a purified population of "bait" cells for the standard method, restricting its use to study specific cell types in complex tissues or clinical samples.	Identifying unique gene expression changes in cells interacting with HER2^+^ bladder cancer cells among CD4^+^ and CD8^+^ T cells.	[66] [71]
	gLCCC	Expresses a synthetic receptor system (synNotch) to observe cell-cell interactions.	A synthetic Notch system can permanently label in vivo cell-cell contact histories, enabling the study of dynamic interactions.	Limited to genetically pre-defined sender and receiver cells, precluding the detection of interactions in unmodified systems.	Monitoring and tracing dynamic cell-cell contacts across diverse in vivo contexts, including embryonic development, tissue homeostasis, and tumor growth in mice.	[72]
RNA	RNA-centric (identifying the proteins interacting or proximal with an RNA of interest)					
	TRIBE	ADAR deaminase fused with a protein of interest (POI), mediating A-to-I editing of interacting RNAs.	Does not require antibody purification and crosslinking.	Exogenous RBP expression can cause artifacts, and RNA tagging exhibits 3′-end bias.	Identifying the in vivo RNA interactome of specific RNA-binding proteins.	[75]
	APEX-RIP/Proximity-Clip	APEX is coupled with RNA immunoprecipitation (RIP) or crosslinking and immunoprecipitation (CLIP) methodologies.	It bypasses antibody purification, recovers localized RNAs, and uses UV crosslinking for direct interactors.	UV crosslinking enhances specificity at the cost of efficiency and necessitates IP-grade antibodies or genetic tagging.	Mapping the spatial RNA-protein interactome.	[76] [77]
RNA	RaPID	Aptamer-recruited biotinylation (MS2-BioID/MS-APEX2)	Avoids crosslinking artifacts and enables direct in vivo biotinylation of proximal proteins.	Requires RNA tagging, may capture indirect interactors.	Working with exogenous RNA, identifies RBPs associated with an RNA of interest.	[28] [78]
	TAPRIP	Utilizes CIRTS3 RNA-targeting system fused to miniTurbo to biotinylate proteins interacting with specific RNA structures.	High-efficiency, biocompatible mapping of RNA-protein interactomes without requiring RNA extraction or crosslinking.	Limited by the delivery efficiency of CIRTS3-miniTurbo system and non-specific labeling due to miniTurbo’s basal activity.	Profiling protein interactomes of non-coding RNAs (e.g., circRNAs) and functional RNA structures (e.g., G-quadruplexes) in live cells.	[79]
	CBRPP	A dCas13-PBL enzyme (e.g., TurboID or APEX) fusion to be guided to a specific endogenous RNA sequence to biotinylate proximal proteins.	High specificity in living cells, eliminating the need for cross-linking or in vitro manipulation.	Limited to the efficient expression and delivery of the large dCas13-PBL fusion construct, which can challenge cellular viability and require optimized delivery systems.	Identifying proteins that interact with or are in proximity to specific non-coding RNAs or mRNA transcripts.	[80]
	Protein-centric (identifying the RNAs interacting or proximal with a POI)					
	APEX-seq	Direct RNA biotinylation, no crosslinking	Fast (~1 min), high spatial efficiency, and is amenable to in vivo applications.	It cannot distinguish direct from indirect interactions, and the biotin-phenoxyl radicals may compromise RNA integrity.	Characterizing the RNA composition of specific regions.	[81] [82]
	CAP-seq	Light-activated proximity-dependent photo-oxidation of RNA nucleobases.	High spatial specificity in live cells.	Requires genetic engineering to express the photosensitizer fusion protein, limiting its application in primary samples.	Investigating subcellular local transcriptomes.	[83]
	dCas13-APEX2/CRUIS	PL enzyme (PafA, BioID, BASU, APEX2) fuses with catalytically inactive dCas13 to biotinylate proteins interacting with an endogenous transcript.	High-specificity, genetic-code-guided mapping of the proteome and transcriptome surrounding specific endogenous RNA.	Requires transfection/expression of multiple large constructs, potential off-target effects.	Identifying proteins and RNAs that interact with or are near nuclear or cytoplasmic RNAs, such as lncRNAs, mRNAs, or viral RNAs.	[84] [85]
	PARIS	UV-activated psoralen to crosslink base-paired RNAs, which are then ligated and sequenced to map interactions.	Achieving of near-base pair resolution in determining RNA structures and interactions directly in living cells.	The requirement for psoralen treatment and UV irradiation, which can be cytotoxic and perturb cellular physiology.	Mapping RNA secondary structures and interaction networks in vivo.	[86]
	Halo-seq	A photoactivatable molecule to label proximal RNAs, which are then purified via click chemistry and sequencing.	Higher efficiency in analyzing small, precisely localized RNA populations (e.g., in suborganellar regions).	Requires precise optical control and might have limited penetration depth in thicker tissues or entire organisms.	Mapping and quantifying RNA localization and the dynamics.	[39]
DNA	DNA-centric (identifying proteins associated with a specific DNA sequence)					
	GLoPro	Peroxidase, dCas9-guided targeting and APEX2-mediated catalytic labeling to biotinylate proteins proximal to a specific genomic locus.	High-specificity, CRISPR-guided profiling of locus-specific protein complexes without crosslinking or harsh extraction.	Limited by dCas9 efficiency and specificity, and by APEX2’s small labeling radius which may miss larger complexes.	Mapping dynamic protein recruitment and regulatory complexes at specific genomic loci (e.g., enhancers, promoters) in native chromatin contexts.	[87]
	CASPEX/C-BERST	Combining CRISPR-dCas9 targeting with APEX2 to biotinylate proteins associated with a defined genomic sequence in live cells.	Enables rapid, unbiased, and high-throughput profiling of proteomes at specific genomic loci without crosslinking or genetic manipulation of endogenous loci.	Limited by the off-target potential of dCas9/gRNA and a small labeling radius that can miss functional distal interactions.	Defining the subnuclear proteome at repetitive genomic elements (e.g., telomeres, centromeres) to annotate novel regulatory factors.	[88] [89]
	CAPTURE	Utilizing a biotinylated, nuclease-deficient Cas9 (dCas9) guided by sgRNAs to target specific genomic loci.	High-resolution, unbiased identification of protein complexes and long-range interactions at a single-copy genomic locus in its native chromatin context.	Limited by the efficient delivery and expression of the large dCas9-sgRNA machinery, and may perturb native cell physiology.	Mapping the proteomic composition and chromatin interactome of specific regulatory elements, such as telomeres, enhancers, and disease-associated loci.	[90]
	ChromID	Biotin ligase, fusing engineered chromatin readers (eCRs) to BASU to label proteins proximal to specific chromatin modifications.	Enables high-specificity mapping of protein interactomes at defined epigenetic marks in living cells.	Requires customized synthetic readers (eCRs), potentially limiting broad applicability and introducing artifacts.	Identifying chromatin-dependent protein interactome and applied to distinct chromatin modifications in mouse stem cells.	[91]
	SelectID	A dCas9-methylation binding domain-TurboID fusion to biotinylate proteins at specific methylated DNA loci.	Enables highly specific proteomic profiling of specific DNA sequences with defined epigenetic marks.	Limited by chromatin accessibility and off-target binding	Discovering novel regulators and readers at specific methylated genomic elements.	[92]
	Protein-centric (identifying the DNAs associated with a POI)					
	ALaP-seq	APEX2 is fused to a POI to detect associated DNA.	Direct, high-resolution mapping of chromatin interactions with nuclear compartments in live cells.	Lower specificity than ChIP-seq, inability to distinguish direct vs. indirect tethering.	Uncovering spatial transcriptional hubs and dissect epigenetic exclusion mechanisms.	[94]
	DamID-seq	Fusing Dam methyltransferase and a DNA-binding protein to methylate adenines in GATC motifs near protein-DNA interaction sites.	Does not require antibodies or crosslinking, providing high-resolution genome-wide mapping of protein-DNA interactions with high sensitivity.	Limited by Dam methyltransferase specificity and the requirement for genetic fusion.	Mapping genome-nuclear lamina interactions and other protein-DNA binding events in living cells with high precision and sensitivity.	[95]
Small molecule	Molecular landscape					
	Photo-activated PL (PAPL)	Photocatalytic, generates reactive species that label lipids upon light activation.	Enables organelle-selective analysis of lipid transport and composition.	Requires introduction of exogenous components and may perturb native systems.	Quantitatively mapping lipid transport and metabolism between specific organelles.	[50]
	Small molecule-Protein interactions					
	DrugID	Biotin ligase, conjugates a drug to a biotin ligase to identify its binding and proximal proteins in live cells.	Mapping drug interactomes de novo in live cells at physiological doses with minimal material.	The drug-enzyme conjugate may alter the native behavior of the drug.	Mapping in vivo drug interactomes, including small molecules (e.g., JQ1) and oligonucleotides (e.g., ASOs).	[96]
	PROCID	HaloTag-mediated proximity biotinylation for target identification.	Enables direct, live-cell mapping of dynamic drug-protein interactions with high specificity.	Requires chemical conjugation of small molecules to HaloTag ligands, may miss transient interactions.	Identifying unknown drug targets and binding sites in live cells, accelerating drug mechanism studies.	[97]
	BioTAC	Biotin ligase, a small-molecule-guided platform to identify proximate proteins in living cells.	In-situ identification of both direct and complex binding proteins within native cellular environments.	Limited by background signal and chimera-induced functional alterations.	Mapping the cellular interactomes of therapeutic small molecules to elucidate mechanisms of action.	[98]
	POST-IT	HaloTag-fused Pup ligase to directly transfer Pup to proximal proteins upon binding to a small molecule in live cells.	Enables non-diffusive, highly specific tagging of drug-protein interactions in live cells, overcoming limitations of lysate-based methods.	Requires optimization to prevent self-pupylation and achieve efficient small-molecule conjugation.	Identifying novel drug targets and off-targets in native physiological contexts, including complex in vivo models (zebrafish embryos).	[99]

#### Mapping dynamic proteomes and interactomes in living systems

A primary application of PL is the characterization of local proteomes and PPI networks within living systems. By capturing biotinylated proteins for mass spectrometry, PL can define the molecular composition of subcellular compartments, membrane contact sites, and transient signaling complexes that are inaccessible to conventional methods [51]. Comprehensive summaries are available elsewhere [16, 19, 34, 52].

Key innovations have significantly expanded the scope and context of proteomic mapping. Antibody-based PL techniques (e.g., BAR [33], AMAPEX [53, 54]) enable interactome discovery in fixed cells and primary human tissues without the need for genetic manipulation. PL also excels in mapping dynamic post-translational modifications (PTMs) [55], using strategies ranging from photoactivatable TurboID/BioID-based profiling of phosphorylation [56, 57] and glycosylation to direct enzymatic labeling of PTMs like O-GlcNAcylation in live cells [58–60]. For dynamic, in vivo applications, engineered enzymes like TyroID can map extracellular proteomes, such as brain region-specific surfaceomes [61], while photoactivated platforms (e.g., miniSOG [36], HaloMap [40]) provide exceptional spatiotemporal control for processes like SG assembly. Furthermore, activity-dependent systems (such as calcium-dependent BioID [Cal-ID]) create permanent, transcription-independent records of transient signaling events, such as calcium signals and neuronal activity [62].

PL can also be integrated with other technologies. CLEM (correlative light and electron microscopy) platforms, such as fast light-EM examination, leverage the electron-dense precipitates generated by PL enzymes (e.g., APEX2), enabling seamless correlation of dynamic tracking of proteins in living cells with the high-resolution ultrastructural mapping within the native cellular architecture [31, 63]. Combining with synchronized trafficking systems, such as the retention using selective hooks (RUSH) system, enables the capture of transient interactors for cargo proteins along the secretory pathway. This approach maps their trafficking interactome for targets like SARS-CoV-2 proteins and lysosomal enzymes under physiological and pathological conditions [64, 65].

PL technologies offer a powerful approach to dissecting cell-cell interactions, ranging from stable adhesions to transient signaling events [66]. They have varying spatial resolutions depending on the operation principles. Diffusible labeling systems such as QMID (quinone methide-assisted identification of cell spatial organization) enable micrometer-scale mapping of cellular neighborhoods and indirect communication [67], while receptor-engagement detectors like LIPSTIC (Labelling Immune Partnerships by SorTagging Intercellular Contacts) provide single-cell resolution of specific ligand-receptor interactions in real-time [68]. For tracing protein exchange between cells, sequential enzymatic labeling approaches including TransitID offer nanometer-scale precision [69]. Recent innovations include self-sufficient peroxidase systems that generate H_2_O_2_ endogenously, enabling sensitive recording of cell-cell contacts under physiological conditions, which is ideal for neural and immune studies [70]. The toolkit further expands to include engineered enzyme systems (FucoID) for identifying specific interaction pairs [71] and synthetic biology platforms (gLCCC) that permanently record contact histories between defined cellular partners [72]. Together, these complementary strategies enable comprehensive mapping of cell-cell communications, bridging nanoscale interaction events to macroscopic cellular behaviors and their resultant functional phenotypes.

#### Resolving RNA-protein interactions and local transcriptomes

PL has revolutionized the study of RNA biology by enabling the mapping of RNA-protein interactions and subcellular RNA localization in living cells, using RNA-centric and protein-centric strategies.

The RNA-centric strategy is designed to identify proteins associated with specific RNA molecules, yielding precise views of RNA-binding protein (RBP) networks [73, 74]. Techniques like TRIBE (Targets of RNA-binding proteins Identified By Editing) use RNA editing enzymes to mark the bound transcripts [75]. These editing sites are then identified through high-throughput sequencing, revealing the targets of the RBP. APEX-RIP/Proximity-CLIP [76, 77] and RaPID [28, 78]/TAPRIP [79] employ proximity biotinylation, with or without crosslinking, to capture RNA-binding proteins. Extending to endogenous transcripts, CBRPP (CRISPR-based RNA proximity proteomics) targets native RNAs directly based on the fusion of dCas13 to PL enzyme [80].

The protein-centric methods, such as APEX-seq and CAP-seq, use peroxidase- or photosensitizer-based labeling to identify all RNAs near a bait protein or organelle, enabling transcriptome-wide mapping of RNA localization with high spatial and temporal resolution [81–83]. Several techniques enable the precise investigation of specific RNAs. For example, dCas13-APEX2/CRUIS (CRISPR-based RNA-United Interacting System) has been used to profile the proximal proteome near telomerase RNA and hTR [84, 85]. Additional modalities like PARIS (Psoralen Analysis of RNA Interactions and Structures) [86] and Halo-seq [39] further enable the study of RNA structures and the dynamics of precisely localized RNA populations. Collectively, these tools have uncovered fundamental principles of RNA regulation and compartmentalization.

#### Profiling chromatin-associated proteomes and genomic landscapes

PL enables high-resolution profiling of the protein complexes associated with specific genomic loci and chromatin states in live cells. CRISPR/dCas9-guided systems such as GLoPro (genomic locus proteomics) [87], C-BERST (dCas9-APEX2 Biotinylation at genomic Elements by Restricted Spatial Tagging) [88, 89], and CAPTURE (CRISPR affinity purification in situ of regulatory elements) [90], which fuse a PL enzyme to dCas9, can direct the PL enzyme to defined DNA sequences, biotinylating proximal proteins, thereby revealing the proteomic landscape of promoters, enhancers, and repetitive elements. Complementing this, chromatin reader-based approaches, such as ChromID (chromatin-dependent protein identification) [91] and SelectID (selective profiling of epigenetic control at genome targets identified by dCas9) [92], map interactomes at specific epigenetic marks. Beyond protein-centric mapping, photosensitizer protein-based labeling enables crosslink-free profiling of chromatin architecture. This method revealed lamina-associated domains that covered ~37.6% of the genome in the nuclear lamina of human embryonic kidney 293 T cells [93]. Meanwhile, ALaP-seq [94] and methylation-based techniques (DamID-seq) [95] have been used to identify DNA regions associated with a nuclear bait protein or to map genome-wide protein-DNA interactions, respectively. These methods have advanced our understanding of nuclear architecture and gene regulation, though challenges remain in minimizing steric interference and improving resolution for closely spaced genomic features.

#### Charting metabolic networks and drug interactomes

PL is being increasingly applied to map small-molecule interactomes. Photo-activated PL (PAPL) generates reactive species to label proximal lipids, enabling organelle-selective analysis of lipid composition and transport [50]. For mapping drug-protein interactions, enzyme-conjugation strategies (e.g., DrugID [96], PROCID [97]) employ covalent conjugation of a drug with a labeling enzyme, allowing unbiased identification of binding and proximal proteins in live cells. Newer platforms like BioTAC (biotin targeting chimera) [98] and POST-IT (Pup-On-target for Small molecule Target Identification Technology) [99] further enhance specificity by minimizing diffusion-driven background, enabling highly precise target identification in physiological contexts and accelerating drug mechanism-of-action studies.

Taken together, these diverse and innovative PL modalities provide an unparalleled toolkit for constructing multi-omic molecular maps in living systems. They enable interrogation of patiotemporal dynamics of interactions across all major biomolecular classes in vivo, advancing our understanding of cellular processes and disease mechanisms.

## Construction of molecular atlas in the nervous system

The cellular diversity and intricate subcellular architecture have long complicated molecular profiling in the CNS. While the single-cell RNA-seq technology resolves neuronal subtypes, and Cre-lox strategies enable gene manipulation [100], these approaches cannot fully capture proteomes within intact subcellular compartments or at cell-cell interfaces. Traditional biochemical fractionation disrupts delicate structures [101], and physical isolation methods, such as laser-capture microdissection (LCM) or fluorescence-activated cell sorting (FACS), can erode morphological context and dilute transient interactions [102, 103]. PL technologies overcome these barriers by enabling high-resolution, organelle-, cell-type-, and interface-specific molecular mapping within native environments.

### Mapping organelle proteomes and interactions

PL has been instrumental in mapping the molecular composition and dynamic interactions of intracellular organelles. For mitochondria and ER, engineered enzymes such as APEX2 and TurboID enable spatially restricted labeling of organelle proteomes [104–106]. Interactome studies in non-neuronal models have identified RTN4IP1 (reticulon 4-interacting protein 1, also known as optic atrophy-10, OPA10) as a crucial mitochondrial oxidoreductase essential for coenzyme Q synthesis [107]. Studies using the split-TurboID tool and peroxidase-mediated proximity biotinylation, respectively, have mapped protein composition of ER-mitochondria contact sites (e.g., involving ABCD3 and LBR, SYNJ2BP-RRBP1) in HEK 293 T cells [108, 109]. These findings provide an essential molecular framework for investigating energy metabolism and organelle communication in neurons, and identifying processes critical for neurotransmission and neuronal survival.

Within the ER, APEX labeling has revealed structures like PERK/COPII-dependent ER whorls that modulate translation during stress [110]. Peroxidase (HRP/APEX2)-based mapping has revealed stress-induced protein mistargeting, including ATF6-dependent ER-import defects [111]. In situ secretory protein labeling via ER-anchored TurboID (iSLET) has enabled dynamic tracking of tissue-specific secretory proteins in the mouse liver [112]. This implies that iSLET is a potential tool for studying secretion of neuropeptides and neuromodulators. For RNA-protein interactomes, TRIBE has identified neuron-specific targets of RNA-binding proteins [74, 113], and the discovery of ER-localized glycoRNAs has challenged conventional models of the secretory pathway [114].

Lysosome-focused PL approaches (e.g., LAMP1-APEX, Lyso-TurboID) have uncovered novel membrane proteins (e.g., SCAMP3, a secretory carrier-associated membrane protein) [115, 116] and repair mechanisms such as the phosphoinositide-initiated membrane tethering and lipid transport (PITT) pathway [117] and TBC1D15-driven tubulation [118] in various cell models. While these studies were performed in non-neuronal models, they are highly relevant to neuroscience, as lysosomal integrity is critically implicated in neurodegeneration [116, 119]. In neurons, ER-lysosome contact mapping has demonstrated that P180 and kinesin-1 directly regulate axonal lysosome trafficking in neurons [120], a finding that directly links PL-defined organelle interactions to neuronal-specific functions.

PL has also illuminated the proteomic architecture of membraneless compartments. CAP-seq mapped SG transcriptomes, revealing enrichment of AU-rich RNAs [121], while APEX2-based SG proteomics identified core constituents such as G3BP1 and autophagy-related factors implicated in amyotrophic lateral sclerosis (ALS) [122]. Critically, in neuronal subcompartments, PL has identified key organizers like SCRIB [123, 124] in the axon initial segment (AIS), and Pdlim7 as a functional component of the spines [125]. These findings provide mechanistic insights into polarized trafficking and synaptic dysfunction. In addition, TurboID labeling proteomics has identified myelin basic protein (MBP) proximity interactions with adhesion proteins, solute carriers, vesicle-transport machinery, and ferroptosis-linked metabolic regulators, suggesting broader roles of MBP in myelin integrity [126].

Collectively, the unique advantages of PL, including its tunable spatial resolution (achieved by varying labeling radii through enzyme engineering or time-controlled activation), precise temporal control, and broad system compatibility (as demonstrated in diverse models such as iPSC-derived neurons [116] and primary tissues [108]), have advanced our understanding of organelle interplay, underscoring the promise of integrated spatial omics for decoding neuronal function and pathology.

### Cell-type-specific molecular census

PL systems are increasingly tailored for cell-type-specific profiling within the nervous system. For neuronal subtypes, LOV-TurboID enables light-controlled, high-precision labeling in the mouse brain and cultured neurons [21]. BioID and TurboID have been used to map interactomes of neurotransmitter receptors (e.g., AMPA receptor [AMPAR], γ-aminobutyric acid sub-type A receptor), revealing developmental shifts in receptor-associated partners [127]. Chemogenetic activation coupled with APEX2 proximity labeling in striatal neurons has uncovered activity-dependent proteome remodeling [128, 129]. In dopaminergic neurons, TurboID identified Kv7.2/7.3 channels as interactors of dopamine and glutamate transporters, suggesting their roles in modulating neurotransmitter uptake relevant to Parkinson’s disease (PD) and epilepsy [130]. Adeno-associated viruse (AAV)-mediated, cell-specific TurboID delivery has facilitated comprehensive proteomic profiling of neurons and astrocytes, identifying approximately 10,000 unique proteins with high reproducibility [131].

PL has also facilitated circuit mapping and activity recording. A study using Cre-dependent APEX2 proximity labeling in corticostriatal neurons has profiled axonal proteome dynamics across postnatal development, linking altered kinase activity to neurodevelopmental phenotypes [129]. Cal-ID, an enzyme that biotinylates nearby proteins within minutes in response to elevated local calcium levels, has been used to record neuronal activity with high spatial resolution and molecular specifivity [62]. Irala et al. [132] used TurboID fused to neurocan (NCAN) termini to delineate distinct cortical interactomes in vivo, showing that the NCAN C-terminus promotes inhibitory synapse formation.

Regarding synapse organization and intercellular communication, a study using BioID in Cx36-EGFP mice and zebrafish uncovered >50 previously unrecognized proteins at electrical synapses, including the scaffold protein Sipa1l3, which shapes synaptic connectivity [133]. Pupylation-based interaction labeling (PUPIL) has been used to map gap junction networks in the mouse brain, offering a non-invasive approach to study neural connectivity [134]. At chemical synapses, a PL study using PSD-95-fused and gephyrin-fused BirA (an *E.coli* biotinylation enzyme) to target the proteomes of excitatory and inhibitory postsynapses, respectively, comprehensively defined 121 excitatory and 181 inhibitory postsynaptic proteins, revealing new regulators of trafficking and signaling [18]. In a notable application, BAR was used to define the physiological interactome of pS129-alpha-synuclein in the healthy mouse olfactory bulb [135]. The identified partners, enriched for presynaptic functions, reposition this pathological epitope as part of normal biology and point to a potentially vulnerable circuit in synucleinopathies. A low-toxicity tyrosine-labeling system (BmTyr) efficiently mapped endogenous interactomes of metabotropic glutamate receptor (Grm1) and dopamine receptor (Drd2) in the mouse brain via ligand-tethered PL [136].

Beyond neurons, PL has illuminated glial signaling. In BV-2 microglia, TurboID fused to Kv1.3 revealed distinct N- and C-terminal interactomes under inflammatory conditions, implicating pathways involving STAT1 and Toll-like receptor (TLR) 2 [137]. A BioID-based study showed that the LPS-induced Neu1 translocation sustains neuroinflammation by desialylating TLR4, highlighting a potential therapeutic target [138]. TurboID profiling in microglial and neuronal lines has revealed native-state proteomes (~60% coverage) with distinct inflammatory signatures, offering systems-level views of neuroinflammatory responses [139].

Together, PL is a powerful tool to resolve cell-type-specific proteomes, elucidate synaptic organization, and uncover disease-relevant mechanisms across neural systems.

### Cell-cell interaction mapping

PL has revolutionized proteomic mapping at cell-cell interfaces within the nervous system, revealing molecular foundations for synaptic and neuro-glial communication. Using spatially restricted enzymatic tagging, Loh et al. profiled excitatory and inhibitory synaptic cleft proteomes with >89% specificity, identifying Mdga2 as a regulator of Neuroligin-2 at inhibitory synapses [17]. Earlier HRP-fusion work targeting synaptic adhesion molecules (e.g., SynCAM1) identified 39 excitatory cleft proteins, including Neuroligin-3 and Neurexin-1, alongside novel candidates such as receptor tyrosine-protein phosphatase zeta [140].

Subsequent innovations enabled systematic dissection of neuro-glial contacts. The Split-TurboID system adapted to astrocyte-neuron interfaces has enabled in vivo labeling at astrocyte-synapse junctions and revealed previously unappreciated protein networks [48, 141]. Another study combining cell type-specific in vivo biotinylation of proteins (CIBOP) and viral delivery produced cell-type-resolved maps of neuronal and astrocytic proteomes with regional specificity, revealing proteomic differences between Camk2a-positive neurons and Aldh1l1-positive astrocytes [141, 142]. BioID2-based protocols enable detailed subcellular proteomics in intact astrocytes and neurons while preserving the morphology [62, 142]. In disease models, HRP-based PL in 5×FAD mice revealed Aβ accumulation at the myelin-axon interface and disrupted signaling pathways linked to lipid metabolism and axonal outgrowth [143].

Collectively, PL has become an indispensable approach to decoding the molecular architecture of neural cell-cell interfaces under physiological and pathological conditions.

## Applications in neurological disease research

**Fig. 3:**
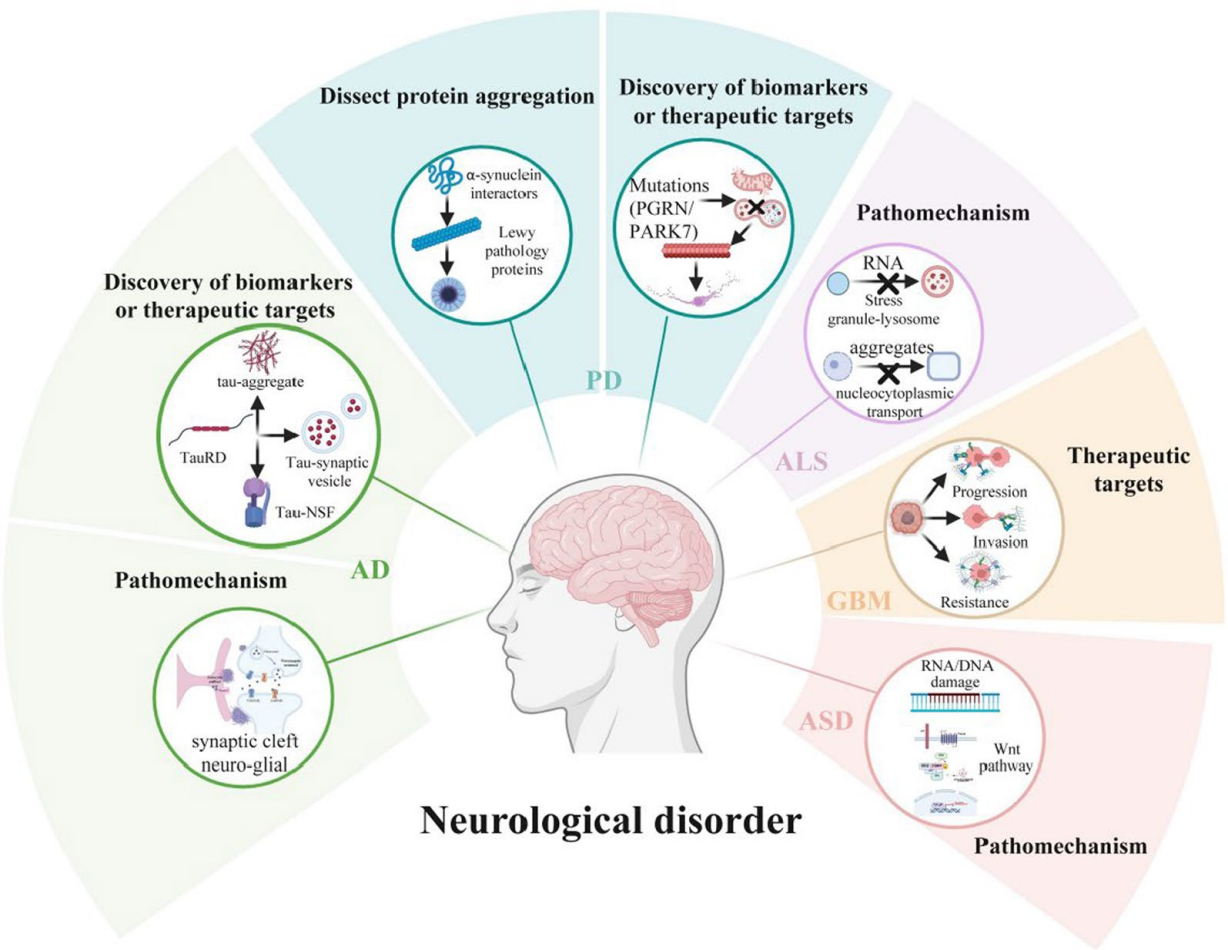
PL-based approaches enable systematic investigation of disease-specific mechanisms, proteomic landscapes-guided biomarkers and druggable targets across major neurological disorders, including Alzheimer’s disease (AD), Parkinson’s disease (PD, ALS, glioblastoma (GBM), and autism spectrum disorder (ASD). Created with BioRender.com

The complexity and inaccessibility of the human nervous system have long impeded a mechanistic understanding of neurological diseases. Traditional methods often fail to capture the spatiotemporal dynamics of molecular events that drive the pathogenesis. PL is transforming neurological disorder research by pinpointing the precise subcellular context and interactors of pathogenic proteins, mapping organelle- and circuit-specific dysfunctions that underlie cellular vulnerability, and directly nominating mechanistic biomarkers and druggable targets (Fig. 3, Table 2).

**Table 2:** Advances in neurological disorders by PL technologies

Disease	Approach	Target	Sample	Main finding	Refs.
AD	split TurboID	Tau microtubule repeat domain (TauRD)	HEK293 cells	Identified 700+ TauRD interactors (RNA-binding/spliceosome proteins) linking nuclear RNA processing to tau pathology.	[144]
	APEX	Human tau	Human iPSC-derived neurons	Identified activity-dependent tau-synaptic vesicle interactions, mapping binding to vesicle cytosolic domains.	[145]
	BioID2	Tau	Primary neurons, mouse brains	Identified tau-NSF interactions regulating AMPAR trafficking and synaptic plasticity underlying memory.	[146]
	Turbo	*O*-GlcNAcylation	*Drosophila* mushroom body	*O*-GlcNAcylation impacts cognitive function via regulating regional translational activity in the brain.	[147]
	HRP	Dystrophic neurites (spheroid)	Human postmortem and mouse brains	Revealed mTOR-mediated protein turnover and cytoskeletal dysregulation in dystrophic neurites drives spheroid pathology.	[148]
	HRP	Myelin-axon interface	Human brains and 5×FAD mice	Demonstrated Aβ accumulation at myelin-axon interfaces disrupts neuro-glial signaling and paranode structure.	[143]
	ProPPr	Phospho‑tau	FFPE tauopathy brain tissues	Cross-disease tau-aggregate proteomics identified disease-specific interactors (VPS35/LAMP2/GSK3α) and a role of microglial ferritin in CBD.	[170]
	CIBOP	Parvalbumin interneurons (PV-INs)	Human brains and 5×FAD mice	PV interneuron proteomics linked mitochondrial/synaptic/mTOR dysfunction to early AD, revealing risk/resilience-associated proteins.	[172]
PD	TurboID	Histone H3.3	HEK293T cells	PARK7 translocates to chromatin upon Cys106 ligation.	[173]
	APEX2	Dopaminergic neurons	Mouse brains	Dopaminergic neuron proteome mapping revealed axonal localization of key ion channels.	[174]
	APEX, BAR	Lysosomes	iPSC‑derived neurons and mouse brains	Progranulin deficiency disrupts lysosomal function via v-ATPase/hydrolase/pH dysregulation, impairing proteostasis.	[175]
	UltraID	α-Synuclein	HEK293T cells and iPSC‑derived neurons	Identified 38 α-synuclein interactors (endolysosomal/membrane proteins) supporting membrane-mediated early aggregation.	[166]
	BAR	Total and pathological α-synuclein	Human brains	Identified 261 Lewy pathology proteins (e.g., HBB) and vesicular/mitochondrial dysregulation in synucleinopathies.	[152]
	BAR	α-Synuclein	Human brains	Identified 79 PD/DLB-linked vesicle/SNARE proteins and 3 MSA markers (CBR1/CRYAB/GFAP) associated with metabolic/oxidative pathways.	[171]
ALS	APEX2	Fused in sarcoma (FUS)	HEK293T cells	FUS N-terminal phosphorylation shifts interactome to cytoplasmic mRNA regulators (e.g., MOV10) and nuclear DNA repair factors, disrupting RNA homeostasis.	[154]
	APEX	Superoxide dismutase 1(SOD 1)	HEK293T cells and N2a cells	ALS-mutant SOD1 (G85R/G93A) aberrantly binds XPO5, disrupting pre-miRNA export and Dicer processing to impair miRNA biogenesis.	[156]
	APEX	Profilin 1 (PFN1)	HEK293T cells and N2a cells	ALS-mutant PFN1 (C71G/M114T) binds splicing factors (hnRNPC/U2AF2), inducing pathogenic Alu exonization.	[155]
	APEX2	Tubulin-binding protein STMN2	U2OS cells and iPSC-derived neurons	STMN2 neuroprotection in ALS involves dynamic tubulin/membrane-state switching at trans-Golgi networks.	[158]
	APEX2	LAMP1-positive lysosomes	Human iPSC-derived neurons	ANXA11 mediates RNA granule-lysosome tethering, a transport mechanism disrupted by ALS mutations.	[159]
	APEX2	Stress granules (SG)	Human iPSC‑derived neurons, *Drosophila*	Identified 150 novel SG components with cell-type-specific profiles, showing neuronal SGs are uniquely enriched in chaperones/autophagy factors.	[122]
	BioID	TDP-43	Human brains, iPSC‑derived neurons, Neuro-2A	TDP-43 aggregates sequester nuclear pore components (Nups/TFs), disrupting nucleocytoplasmic transport.	[154]
	BioID2	C9orf72-derived dipeptide repeat proteins	Human postmortem brains	C9orf72 DPR interactomes revealed polyGA aggregates sequester VCP, impairing autophagy in ALS/FTLD.	[168]
ASD	TurboID	DDX3X	Mouse primary neural progenitors	DDX3X mutations impair neurodevelopment through RNA/DNA damage pathways, with severe cases causing neuronal death.	[160]
	BioID2	Synapsin1	Mouse primary neurons	Identified 41 ASD gene networks disrupting mitochondrial/Wnt/MAPK pathways and correlating with clinical severity.	[161]
GBM	BioID2	Epithelial membrane protein 3 (EMP3)	U-118 glioblastoma cells	EMP3 sustains oncogenic EGFR/CDK2 signaling in GBM by blocking RAB7-mediated degradation via TBC1D5, driving proliferation and therapy resistance.	[176]
	PAPL	EGFR	Glioblastoma tissue, GBM43 cells	Live-cell and GBM tissue EGFR interactomes unveiled spatially distinct microenvironment interactions.	[177]
	Turbo-ID	ACP1, AURKB	GBM43 cells	Cortactin links ACP1-AURKB in GBM invasion via serine-phospho/tyrosine-dephospho regulation.	[178]

### Elucidating disease mechanisms with spatiotemporal precision

In Alzheimer’s disease (AD), PL has revolutionized the study of tau and Aβ pathology. In a study dissecting the role of tau, split-TurboID identified over 700 interactors of the microtubule-binding repeat domain of tau (TauRD), which demonstrate a surprising enrichment for RNA-binding and spliceosome proteins. This finding implicates a novel mechanism where tau disrupts nuclear pathways beyond its classical role in microtubule stability [144]. Complementary APEX-based labeling in human iPSC-derived neurons has mapped tau interactions with synaptic-vesicle proteins during activity-dependent tau secretion, providing a potential mechanism for activity-regulated tau secretion and spread [145]. Furthermore, BioID2 uncovered an interaction between tau and N-ethylmaleimide-sensitive factor (NSF), which regulates AMPAR trafficking. This interaction provides a direct molecular link between tau pathology and the synaptic plasticity deficits underlying memory loss in AD [146]. Notably, a study combining an *O*-GlcNAc binding activity with TurboID proximity labeling in *Drosophila* identified components of the translational machinery as substrates of *O*-GlcNAcylation, suggesting that *O*-GlcNAcylation modulates regional translational activity in the brain and thus impact cognitive function [147]. Beyond tau, HRP-based PL in human post-mortem brains and mouse brains revealed mTOR-driven proteostasis defects and cytoskeletal dysregulation within dystrophic neurites, offering direct insights into the mechanisms of spheroid pathology [148]. Additionally, in HT22 cells, BioID mapping of the beta-site amyloid precursor protein cleaving enzyme 1 (BACE1) interactome, upstream in the amyloid cascade, identified time-resolved proximal proteins, informing the dynamic regulation of amyloidogenic processing [149].

In PD, complex and dynamic interactomes of α-synuclein have been revealed by PL. APEX2 mapping in neurons identified 225 α-synuclein-proximal proteins involved in membrane trafficking and RNA binding, many of which overlap with known PD risk factors [150]. Crucially, PL studies have compared molecular interactions for wild-type versus pathological (e.g., A53T, pSer129) α-synuclein. Analysis of human tissues using the BioID system revealed transcriptional adapter 2-alpha (TADA2α) as a novel binding partner of α-syn, and found a much stronger interaction between α-synuclein A53T and TADA2α than that between wild-type α-synuclein and TADA2α [151]. The BAR technique labeling total and pathological α-synuclein showed disease-specific interactions of pSer129 α-synuclein with vesicular proteins [152]. PAPL extended these findings by enabling organelle-specific profiling and nominating novel Parkin substrates (e.g., Ssu72/SNW1) with relevance to PD pathogenesis [143].

In ALS and frontotemporal dementia, PL has been used to study RNA-binding protein pathology. APEX-based approaches revealed that ALS-linked mutants of SOD1 and profilin 1 (PFN1) have aberrant interactions with the miRNA biogenesis machinery (Exportin 5) and mRNA splicing factors (hnRNPC, U2AF2), respectively. This finding directly connects these mutations to RNA dysregulation [155, 156]. Furthermore, using stress-context-dependent APEX2 proximity mapping in iPSC-derived neurons, we found that the interactions of TDP-43 with specific splicing factors (e.g., SRRM2) within membrane-less organelles were significantly altered. Integrated with single-nucleus RNA-seq, this approach identified NUFIP2 as an interactor with TDP-43 that promotes TDP-43 aggregation and HNRNPC as a TDP-43 splicing regulator that can be modulated to rescue TDP-43-mediated toxicity [157]. PL has also elucidated dynamic molecular shifts underlying ALS pathogenesis. In iPSC-derived neurons, APEX2 mapping revealed that STMN2, a protein critical for neuronal integrity, undergoes a disease-associated shift from the tubulin-bound to the membrane-associated state [158]. PL has also elucidated critical organelle communication defects in ALS. APEX2 mapping identified annexin A11 (ANXA11) as a key tether between RNA granules and lysosomes, a mechanism essential for RNA transport and degradation that is disrupted by ALS-linked *ANXA11* mutations [159].

In autism spectrum disorder (ASD), PL is systematically bridging genetic risk loci to convergent molecular pathways. TurboID in mouse neural progenitors revealed that *DDX3X* mutations cause variant-specific disruptions in RNA metabolism and DNA damage response, with severe mutations leading to neuronal death [160]. A BioID2-based interaction network covering 41 ASD risk genes revealed convergent disruptions in mitochondrial, Wnt, and MAPK signaling pathways, which correlate with clinical severity [161]. Another study using BioID2 identified Ankyrin-G as a major interactor of the deubiquitinase OTUD7A (Ovarian tumor domain-containing protein 7 A), thereby linking proteostasis dysregulation to impaired neuronal development [162]. PL also identified activity-dependent neuroprotective protein (ADNP) as an R-loop suppressor. Dysfunction of ADNP drives R-loop/CTCF accumulation and impairs neuronal differentiation, while zinc-finger/homeodomain proteins were nominated in the study as broader R-loop regulators [163].

Collectively, these studies highlight PL as a powerful tool to reveal early pathogenic events at high spatial and temporal resolutions, including protein mislocalization, RNA dysregulation, and organelle miscommunication.

### Dissecting protein aggregation and proteostasis failure

A paramount strength of PL lies in the ability to resolve the composition and dynamics of pathogenic aggregates and dysfunctional organelles, which are hallmarks of neurodegenerative diseases. Innovative PL methods have been developed to specifically target these processes. AggID, a photo-induced proximity labeling and crosslinking approach, has selectively profiled intracellular aggregates, uncovering HSP70-mediated proteostasis networks and autophagy activation as a response to proteotoxic stress [164]. As mentioned, UltraID-LIPA has provided a temporal map of α-synuclein oligomerization [165], implicating endolysosomal and membrane-associated partners in the initial oligomerization events. Furthermore, the upsFP-tag (conjugating the ultra-photosensitized fluorescent protein chromophore to HaloTag) technology which leverages the radical-mediated labeling mechanism, enabled dynamic interactome profiling of TDP-43 during liquid-liquid phase separation, offering a new tool to study ALS [166]. The AggID and UltraID-LIPA techniques, with the ability to profile the earliest stages of aggregation, provide opportunities for very early therapeutic intervention.

PL is also critical for understanding the functional consequences of aggregation. DenseMAP has resolved SG-specific interactomes of m6A readers (YTHDF1/2) and nucleolar proteomes, highlighting the role of SUMOylation in maintaining nucleolar homeostasis [167]. BioID2 has mapped the interactome of C9orf72-derived dipeptide repeat proteins (DPRs), showing that polyGA aggregates sequester and impair the function of valosin-containing protein (VCP), a key regulator of autophagy, thereby driving neurodegeneration [168]. Similarly, split-APEX has revealed tau interaction with VCP during aggregation, linking impaired protein quality control to AD pathogenesis [169]. These studies move beyond cataloging aggregate constituents to reveal how sequestration disrupts cellular quality control systems.

### Discovering disease-specific biomarkers

By defining in situ, disease-specific interactomes directly in human post-mortem tissues, PL provides a powerful and direct route for biomarker discovery. ProPPr (probe-dependent proximity profiling) applied to formalin-fixed paraffin-embedded (FFPE) tissues of four distinct tauopathies revealed unique tau-aggregate proteomes for each disease. VPS35 (vacuolar protein sorting-associated protein 35) and LAMP2 (lysosome-associated membrane glycoprotein 2) were identified as specific interactors in corticobasal degeneration (CBD). In addition, ferritin light chain (FTL)-positive microglia were revealed to be present in CBD astrocytic plaques. These results offer potential diagnostic markers to differentiate between clinically overlapping tauopathies [170].

In synucleinopathies, BAR has been used to identify 261 proteins associated with Lewy bodies in human brains, with HBB (hemoglobin subunit beta) emerging as a key marker of vesicular and mitochondrial dysfunction in PD and dementia with Lewy bodies (DLB) [142]. A subsequent BAR study further differentiated PD/DLB from multiple system atrophy (MSA), identifying 79 PD/DLB-specific SNARE/vesicle proteins and three MSA-specific markers (CBR1, CRYAB, GFAP) linked to metabolic detoxification pathways [171]. These findings highlight the potential of PL to generate histopathology-defined molecular biomarkers that could improve diagnostic accuracy and stratify patients for targeted therapies.

### Identifying novel therapeutic targets

By mapping disease-relevant protein networks with high precision, PL has accelerated the identification of novel, druggable targets for neurological disorders and brain cancers.

In AD, cell-type-specific PL in 5×FAD mice using the CIBOP system revealed mitochondrial and synaptic proteome dysfunction in parvalbumin interneurons (PV-INs) early in disease, nominating mTOR and related pathways as potential targets for early intervention [172].

In PD, TurboID revealed that the PD-associated protein PARK7/DJ-1 undergoes redox-sensitive nuclear translocation via modification at Cys106, implicating nuclear oxidative stress signaling as a potential therapeutic avenue [173]. Moreover, APEX2 mapping in dopaminergic neurons uncovered aberrant axonal localization of specific ion channels, suggesting new approaches to correcting neuronal excitability defects [174]. Similarly, APEX/BAR PL in iPSC-derived neurons demonstrated that progranulin deficiency disrupts lysosomal function via v-ATPase dysregulation, highlighting lysosomal pH modulation as a potential therapeutic strategy for frontotemporal dementia and other neurodegenerative disorders [175].

In glioblastoma (GBM), PL has been used to map oncogenic signaling networks with therapeutic implication. BioID2 revealed that epithelial membrane protein 3 (EMP3) sustains oncogenic EGFR signaling by interacting with the endocytic regulator TBC1D5 to block RAB7-mediated EGFR degradation, driving proliferation and therapy resistance [176]. This mechanism presents EMP3 and its interactions as a promising target to overcome kinase inhibitor resistance. Another study using red-light-activated photocatalytic PL spatially resolved the EGFR interactome within primary GBM tissues, uncovering tumor microenvironment-specific interactions that could be exploited therapeutically [177]. Furthermore, TurboID identified cortactin as a critical node linking ACP1 and AURKB in GBM invasion, where coordinated phosphorylation/dephosphorylation regulates tumor motility, suggesting dual kinase-phosphatase inhibition as a novel therapeutic approach [178].

Collectively, these advances underscore the capacity of PL not only to map disease-relevant networks at high resolution, but also to directly nominate tractable targets across a wide spectrum of neurological disorders.

## Challenges and future perspectives

While PL has revolutionized molecular mapping in neuroscience, several inherent limitations must be acknowledged. A primary challenge is the spatial resolution of PL. The typical labeling radius (10–20 nm) captures a ‘proximitome’ of proteins that are merely near the bait, complicating the distinction between direct interactors and proximal neighbors. This necessitates orthogonal validation (e.g., Co-IP or surface plasmon resonance) to confirm direct physical interactions. Emerging methods are addressing this core limitation. For instance, SRPL (super-resolution proximity labeling) enhances specificity by minimizing background [179], while other frameworks leverage direct biotinylation site analysis to help distinguish direct binders from proximal neighbors [180]. Despite these advances, further innovation in spatial precision remains a critical future direction.

Other technical hurdles include confounding background signals from endogenous biotin or basal enzyme activity (e.g., TurboID), the substantial size of enzyme tags (~28–35 kDa) which may perturb the native function of bait proteins, and practical constraints for in vivo applications such as limited penetration and substrate delivery, and peroxide-associated toxicity (for APEX2). Furthermore, the analytical pipeline presents its own challenges; large-scale MS datasets demand robust statistical frameworks and meticulously designed controls (e.g., enzyme-alone, catalytically dead, mislocalized mutants) to reliably distinguish true interactors from background noise and establish principled significance thresholds.

**Fig. 4:**
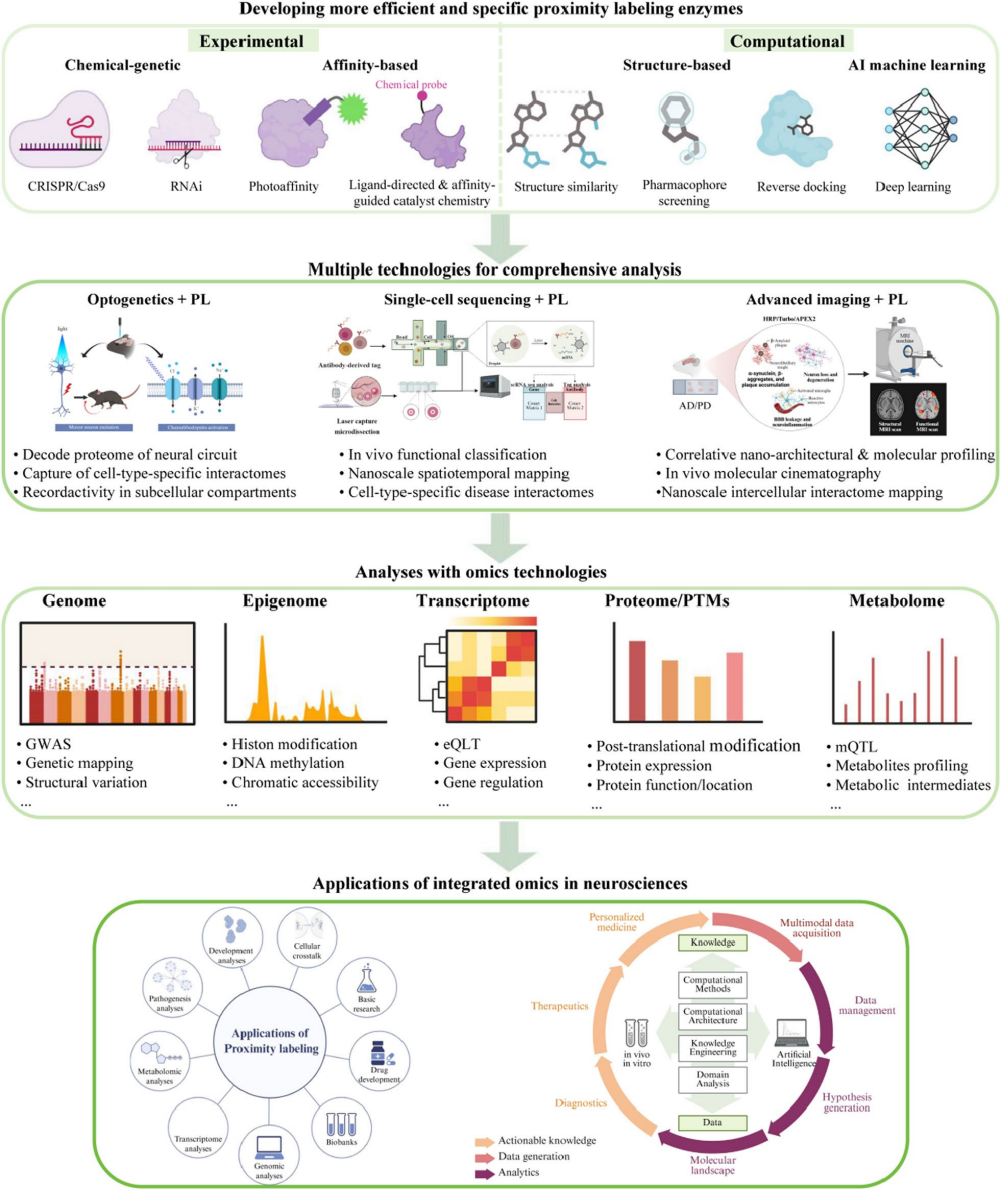
Future directions of PL technologies. Advances in PL will rely on the development of highly efficient and specific enzymes, combined with integration into multi-modal frameworks including optogenetics, single-cell sequencing, advanced imaging, and multi-omics approaches (genomics, transcriptomics, proteomics, PTMs, metabolomics). These innovations will enable deeper investigation into developmental and pathological mechanisms, accelerate biomarker discovery and drug development, and support the integration of multimodal data for mapping comprehensive molecular landscapes in neuroscience. Created with BioRender.com

These limitations, however, are powerful drivers of innovation. The future of PL lies in addressing these challenges through three interconnected avenues (Fig. 4).

1. Precision engineering of next-generation labeling systems

Future progress in PL hinges on a concerted, multidisciplinary effort to engineer next-generation enzymes and labeling chemistries, aimed squarely at overcoming current limitations in specificity, efficiency, and spatiotemporal control. By synergizing computational design (leveraging artificial intelligence and deep learning to predict enzyme mutants with enhanced properties), structural biology (enabling rational design based on enzyme-substrate complexes), and chemical biology (developing novel, membrane-permeant probes and photo-caged substrates), we anticipate the creation of a versatile toolkit of "designer" enzymes.

The key goals of this endeavor include development of smaller tags to minimize bait perturbation, orthogonal enzymes for simultaneous multi-compartment labeling, and conditionally activated enzymes controlled by light (optogenetics) or small molecules for exquisite spatiotemporal control. Concurrently, innovations of advanced probes and substrates will be crucial to enhance specificity, reduce background labeling, and ultimately enable the precise capture of transient, low-affinity, or organelle-specific interactions that are currently beyond reach.

(2) Deep integration with multi-omics and spatial technologies for systems-level interactomics

The full potential of PL will be realized through its deep integration with multi-omics technologies and cutting-edge spatial mapping techniques. This synergy is crucial to transition from static catalogs of interactions to a dynamic, systems-level understanding of molecular networks. For instance, combining cell-type-specific PL with single-cell sequencing can deconvolute the proteomic complexity of heterogeneous tissues like the brain, directly linking genetic variants from genome-wide association studies to local protein interaction networks within specific neuronal populations [181]. Furthermore, correlating PL data with spatial transcriptomics and advanced imaging (e.g., expansion microscopy, cryo-electron tomography) [31, 63] will enable the construction of "molecular cartographies"—comprehensive maps that define protein communities and their relationships within the native nano-architectural context. This integrated approach is pivotal for elucidating the spatial underpinnings of diseases like Alzheimer’s and Parkinson’s, where subtle alterations in local interactomes precede overt pathology.

A parallel and critical frontier is the progression toward single-cell PL proteomics. While bulk PL provides a population-averaged view, emerging methods are pushing the resolution to the single-cell level. Current approaches, such as combining PL with single-cell mass cytometry or carrier-based ultra-sensitive proteomics, are beginning to reveal cell-to-cell heterogeneity in protein proximity networks. The ultimate goal of true single-cell PL-proteomics, which integrates PL, cell sorting or cell capture, and nanoscale proteome analysis, will be indispensable for mapping interactome variations across individual cells within complex tissues. This resolution is crucial for dissecting rare cell types and transient cellular states in development and disease.

The realization of these technological advances, however, is critically limited by a parallel bioinformatic challenge: the current underrepresentation of PL-derived ‘proximitomes’ in canonical protein interaction databases (e.g., STRING, BioGRID and IntAct). This gap stems from the fundamental distinction between ‘proximity’ and ‘direct interaction’, a lack of standardized data formats, and the highly context-dependent nature of PL results. To bridge this divide, a community-driven initiative is essential to create specialized databases or modules that curate PL data as a distinct class of evidence, tagged with rich experimental metadata (e.g., bait, cell type, condition). Establishing such a standardized framework will allow researchers to contextualize new findings, validate PL data against known complexes, and construct unified networks that integrate physical, genetic, and spatial interaction evidence. This bioinformatic integration is the keystone that will transform PL from a powerful discovery tool into a foundational, systems-level resource for biology.

(3) Toward functional and dynamic analysis in living organisms

The ultimate frontier for PL is to evolve from providing static molecular snapshots to recording the real-time dynamics of proteome organization and function within living organisms. Achieving this goal necessitates the integration of PL with technologies that provide direct functional readouts. For instance, combining optogenetics with PL can capture activity-dependent interactome remodeling in stimulated neurons, while coupling PL with metabolic tracing can reveal how metabolic fluxes reshape local protein environments. The development of robust, non-perturbative in vivo PL methods will further enable researchers to track cell-cell communication and the modulation of intercellular interactions in disease models over time. This integrated direction moves beyond merely cataloging "who is there" to understanding "what they are doing" in a physiological context, directly linking molecular proximity to biological function and paving the way for discovering novel functional biomarkers and therapeutic targets.

A central challenge in this endeavor is the functional interpretation of PL-generated candidate lists, specifically the distinction of biologically meaningful interactions from incidental proximity within the labeled ‘proximitome’. Moving from a candidate list to validated biology requires a hierarchical, multi-layered validation strategy. This process begins with bioinformatic triage, where candidates are prioritized using enrichment scores, correlation with public datasets, and pathway analysis. The ensuing biochemical validation phase employs orthogonal methods like Co-IP to confirm direct or stable physical associations for high-confidence candidates.

Underpinning this entire validation roadmap is a rigorous analytical pipeline for data analysis and hit prioritization. The proteomic output of a PL experiment—often encompassing hundreds to thousands of candidates—demands a systematic, multi-layered workflow to distinguish high-confidence proximal proteins from background. This process begins with foundational statistical rigor, involving robust pre-processing, normalization against critical negative controls (e.g., catalytically dead enzymes), and differential abundance analysis to generate a primary list of significantly enriched proteins. Subsequently, an advanced prioritization phase is implemented to overcome common proteomic biases and distinguish biologically meaningful interactions. This includes applying abundance-corrected scoring systems (e.g., SAINT score) to effectively deprioritize ubiquitous proteins like tubulin; filtering candidates based on subcellular context and cross-referencing with external databases to enhance biological plausibility; and conducting functional enrichment analysis to shift focus from individual hits to coherent biological pathways. This comprehensive bioinformatic workflow generates a tractable shortlist of high-confidence candidates, creating a direct bridge to the downstream experimental validation cascade and ensuring a solid foundation for subsequent functional studies.

To definitively establish functional relevance, a suite of cell-based and in vivo assays is paramount. This begins with visually confirming and quantifying the putative interaction in situ using techniques such as proximity ligation assay, fluorescence resonance energy transfer, or bioluminescence resonance energy transfer, complemented by high-resolution microscopy to verify co-localization within the same subcellular compartment. Beyond mere proximity, the functional consequence of the interaction must be assessed through direct perturbation, for instance, using siRNA or CRISPR knockout of the candidate protein, to measure the impact on the localization, stability, or downstream phenotypic outputs of the bait protein. Ultimately, the gold standard for validation is demonstrating the physiological relevance of the interaction within the complex milieu of a living model organism, thereby bridging molecular proximity to tangible biological function.

As these technological and analytical frameworks mature, PL will be uniquely positioned to bridge fundamental neurobiology and clinical translation, transforming spatial hypotheses into mechanistically grounded insights and enabling precision-medicine strategies for neurological diseases.

## Conclusion

PL has become a cornerstone methodology in neuroscience, enabling high-resolution mapping of molecular architecture within specialized neural microdomains, including synaptic clefts, postsynaptic densities, and organelle contact sites. By capturing transient, low-affinity, and spatially confined interactions under near-physiological conditions, PL has revealed previously inaccessible protein networks and time-resolved interactomes, yielding critical insights into normal circuit function and the pathogenesis of neurological disease.

Future promising directions lie in addressing persistent challenges through deep integration. Coupling PL with spatial transcriptomics, single-cell proteomics, and advanced imaging will enable the construction of comprehensive molecular atlases. Furthermore, applying PL directly to human tissues and clinically annotated cohorts holds the potential to yield in situ biomarkers and nominate druggable targets. In sum, by seamlessly bridging fundamental neurobiology and clinical translation, PL is poised to serve as a foundational pillar for precision neurology, advancing our understanding of the nervous system and accelerating development of targeted diagnostics and treatments for a broad spectrum of neurodegenerative and neuropsychiatric disorders.

## Data Availability

Not applicable.

## Abbreviations

AD: Alzheimer’s disease
ALS: Amyotrophic lateral sclerosis
APEX: Ascorbate peroxidase
ASD: Autistic spectrum disorder
BAR: Biotinylation by antibody recognition
CNS: Central nervous system
ER: Endoplasmic reticulum
FTLD: Frontotemporal lobar degeneration
GBM: Glioblastoma
LD: Lipid droplet
MS: Mass spectrometry
PD: Parkinson’s disease
PL: Proximity label
POI: Protein of interest
PPI: Protein-protein interaction
PTMs: Post-translational modifications
RBP: RNA-binding protein
SG: Stress granules

## References

[1] Zeisel A, Hochgerner H, Lönnerberg P, Johnsson A, Memic F, van der Zwan J, et al. Molecular architecture of the mouse nervous system. Cell. 2018;174(4):999-1014.e22. 30096314 10.1016/j.cell.2018.06.021 PMC6086934

[2] van Oostrum M, Schuman EM. Understanding the molecular diversity of synapses. Nat Rev Neurosci. 2025;26(2):65–81. 39638892 10.1038/s41583-024-00888-w

[3] Sultana OF, Bandaru M, Islam MA, Reddy PH. Unraveling the complexity of human brain: structure, function in healthy and disease states. Ageing Res Rev. 2024;100:102414. 39002647 10.1016/j.arr.2024.102414 PMC11384519

[4] Dang V, Voigt B, Marcotte EM. Progress toward a comprehensive brain protein interactome. Biochem Soc Trans. 2025;53(1):303–14. 39936389 10.1042/BST20241135 PMC12203947

[5] Chen Y, Fu AKY, Ip NY. Synaptic dysfunction in Alzheimer’s disease: mechanisms and therapeutic strategies. Pharmacol Ther. 2019;195:186–98. 30439458 10.1016/j.pharmthera.2018.11.006

[6] Barcomb K, Ford CP. Alterations in neurotransmitter co-release in Parkinson’s disease. Exp Neurol. 2023;370:114562. 37802381 10.1016/j.expneurol.2023.114562 PMC10842357

[7] Ochneva A, Zorkina Y, Abramova O, Pavlova O, Ushakova V, Morozova A, et al. Protein misfolding and aggregation in the brain: Common pathogenetic pathways in neurodegenerative and mental disorders. Int J Mol Sci. 2022;23(22):14498. 36430976 10.3390/ijms232214498 PMC9695177

[8] Moffitt JR, Lundberg E, Heyn H. The emerging landscape of spatial profiling technologies. Nat Rev Genet. 2022;23(12):741–59. 35859028 10.1038/s41576-022-00515-3

[9] Unterauer EM, Shetab Boushehri S, Jevdokimenko K, Masullo LA, Ganji M, Sograte-Idrissi S, et al. Spatial proteomics in neurons at single-protein resolution. Cell. 2024;187(7):1785-1800.e16. 38552614 10.1016/j.cell.2024.02.045

[10] Huttlin EL, Bruckner RJ, Paulo JA, Cannon JR, Ting L, Baltier K, et al. Architecture of the human interactome defines protein communities and disease networks. Nature. 2017;545(7655):505–9. 28514442 10.1038/nature22366 PMC5531611

[11] Schwenk J, Baehrens D, Haupt A, Bildl W, Boudkkazi S, Roeper J, et al. Regional diversity and developmental dynamics of the AMPA-receptor proteome in the mammalian brain. Neuron. 2014;84(1):41–54. 25242221 10.1016/j.neuron.2014.08.044

[12] Snider J, Kotlyar M, Saraon P, Yao Z, Jurisica I, Stagljar I. Fundamentals of protein interaction network mapping. Mol Syst Biol. 2015;11(12):848. 26681426 10.15252/msb.20156351 PMC4704491

[13] Roux KJ, Kim DI, Raida M, Burke B. A promiscuous biotin ligase fusion protein identifies proximal and interacting proteins in mammalian cells. J Cell Biol. 2012;196(6):801–10. 22412018 10.1083/jcb.201112098 PMC3308701

[14] Branon TC, Bosch JA, Sanchez AD, Udeshi ND, Svinkina T, Carr SA, et al. Efficient proximity labeling in living cells and organisms with TurboID. Nat Biotechnol. 2018;36(9):880–7. 30125270 10.1038/nbt.4201 PMC6126969

[15] Rhee HW, Zou P, Udeshi ND, Martell JD, Mootha VK, Carr SA, et al. Proteomic mapping of mitochondria in living cells via spatially restricted enzymatic tagging. Science. 2013;339(6125):1328–31. 23371551 10.1126/science.1230593 PMC3916822

[16] Qin W, Cho KF, Cavanagh PE, Ting AY. Deciphering molecular interactions by proximity labeling. Nat Methods. 2021;18(2):133–43. 33432242 10.1038/s41592-020-01010-5 PMC10548357

[17] Loh KH, Stawski PS, Draycott AS, Udeshi ND, Lehrman EK, Wilton DK, et al. Proteomic analysis of unbounded cellular compartments: Synaptic clefts. Cell. 2016;166(5):1295-1307.e21. 27565350 10.1016/j.cell.2016.07.041 PMC5167540

[18] Uezu A, Kanak DJ, Bradshaw TW, Soderblom EJ, Catavero CM, Burette AC, et al. Identification of an elaborate complex mediating postsynaptic inhibition. Science. 2016;353(6304):1123–9. 27609886 10.1126/science.aag0821 PMC5432043

[19] Mathew B, Bathla S, Williams KR, Nairn AC. Deciphering spatial protein-protein interactions in brain using proximity labeling. Mol Cell Proteomics. 2022;21(11):100422. 36198386 10.1016/j.mcpro.2022.100422 PMC9650050

[20] Shuster SA, Li J, Chon U, Sinantha-Hu MC, Luginbuhl DJ, Udeshi ND, et al. In situ cell-type-specific cell-surface proteomic profiling in mice. Neuron. 2022;110(23):3882-3896.e9. 36220098 10.1016/j.neuron.2022.09.025 PMC9742329

[21] Lee SY, Cheah JS, Zhao B, Xu C, Roh H, Kim CK, et al. Engineered allostery in light-regulated LOV-Turbo enables precise spatiotemporal control of proximity labeling in living cells. Nat Methods. 2023;20(6):908–17. 37188954 10.1038/s41592-023-01880-5 PMC10539039

[22] Hananya N, Ye X, Koren S, Muir TW. A genetically encoded photoproximity labeling approach for mapping protein territories. Proc Natl Acad Sci U S A. 2023;120(16):e2219339120. 37036999 10.1073/pnas.2219339120 PMC10120045

[23] Gingras AC, Abe KT, Raught B. Getting to know the neighborhood: using proximity-dependent biotinylation to characterize protein complexes and map organelles. Curr Opin Chem Biol. 2019;48:44–54. 30458335 10.1016/j.cbpa.2018.10.017

[24] Shkel O, Kharkivska Y, Kim YK, Lee JS. Proximity labeling techniques: a multi-omics toolbox. Chem Asian J. 2022;17(2):e202101240. 34850572 10.1002/asia.202101240

[25] Seath CP, Trowbridge AD, Muir TW, MacMillan DWC. Reactive intermediates for interactome mapping. Chem Soc Rev. 2021;50(5):2911–26. 33458734 10.1039/d0cs01366h

[26] Choi CR, Rhee HW. Proximity labeling: an enzymatic tool for spatial biology. Trends Biotechnol. 2022;40(2):145–8. 34663510 10.1016/j.tibtech.2021.09.008

[27] Kim DI, Jensen SC, Noble KA, Kc B, Roux KH, Motamedchaboki K, et al. An improved smaller biotin ligase for BioID proximity labeling. Mol Biol Cell. 2016;27(8):1188–96. 26912792 10.1091/mbc.E15-12-0844 PMC4831873

[28] Ramanathan M, Majzoub K, Rao DS, Neela PH, Zarnegar BJ, Mondal S, et al. RNA-protein interaction detection in living cells. Nat Methods. 2018;15(3):207–12. 29400715 10.1038/nmeth.4601 PMC5886736

[29] Kido K, Yamanaka S, Nakano S, Motani K, Shinohara S, Nozawa A, et al. AirID, a novel proximity biotinylation enzyme, for analysis of protein-protein interactions. Elife. 2020;9:e54983. 32391793 10.7554/eLife.54983 PMC7302878

[30] Martell JD, Deerinck TJ, Sancak Y, Poulos TL, Mootha VK, Sosinsky GE, et al. Engineered ascorbate peroxidase as a genetically encoded reporter for electron microscopy. Nat Biotechnol. 2012;30(11):1143–8. 23086203 10.1038/nbt.2375 PMC3699407

[31] Lam SS, Martell JD, Kamer KJ, Deerinck TJ, Ellisman MH, Mootha VK, et al. Directed evolution of APEX2 for electron microscopy and proximity labeling. Nat Methods. 2015;12(1):51–4. 25419960 10.1038/nmeth.3179 PMC4296904

[32] Kotani N, Gu J, Isaji T, Udaka K, Taniguchi N, Honke K. Biochemical visualization of cell surface molecular clustering in living cells. Proc Natl Acad Sci U S A. 2008;105(21):7405–9. 18495923 10.1073/pnas.0710346105 PMC2396715

[33] Bar DZ, Atkatsh K, Tavarez U, Erdos MR, Gruenbaum Y, Collins FS. Biotinylation by antibody recognition-a method for proximity labeling. Nat Methods. 2018;15(2):127–33. 29256494 10.1038/nmeth.4533 PMC5790613

[34] Lee JG, Jeong I, Kim KE. Bridging molecular and cellular neuroscience with proximity labeling technologies. Exp Mol Med. 2025;57(7):1492–505. 40640545 10.1038/s12276-025-01491-4 PMC12322126

[35] To TL, Medzihradszky KF, Burlingame AL, DeGrado WF, Jo H, Shu X. Photoactivatable protein labeling by singlet oxygen mediated reactions. Bioorg Med Chem Lett. 2016;26(14):3359–63. 27220724 10.1016/j.bmcl.2016.05.034 PMC4903891

[36] Li L, Han J, Lo HG, Tam WWL, Jia H, Tse ECM, et al. Symmetry-breaking malachite green as a near-infrared light-activated fluorogenic photosensitizer for RNA proximity labeling. Nucleic Acids Res. 2024;52(7):e36. 38407347 10.1093/nar/gkae125 PMC11040151

[37] Huang Z, Liu Z, Xie X, Zeng R, Chen Z, Kong L, et al. Bioorthogonal photocatalytic decaging-enabled mitochondrial proteomics. J Am Chem Soc. 2021;143(44):18714–20. 34709827 10.1021/jacs.1c09171

[38] Nakane K, Sato S, Niwa T, Tsushima M, Tomoshige S, Taguchi H, et al. Proximity histidine labeling by umpolung strategy using singlet oxygen. J Am Chem Soc. 2021;143(20):7726–31. 33904715 10.1021/jacs.1c01626

[39] Engel KL, Lo HG, Goering R, Li Y, Spitale RC, Taliaferro JM. Analysis of subcellular transcriptomes by RNA proximity labeling with Halo-seq. Nucleic Acids Res. 2022;50(4):e24. 34875090 10.1093/nar/gkab1185 PMC8887463

[40] Pan CR, Knutson SD, Huth SW, MacMillan DWC. µmap proximity labeling in living cells reveals stress granule disassembly mechanisms. Nat Chem Biol. 2025;21(4):490–500. 39215100 10.1038/s41589-024-01721-2 PMC11868469

[41] Lin Z, Schaefer K, Lui I, Yao Z, Fossati A, Swaney DL, et al. Multiscale photocatalytic proximity labeling reveals cell surface neighbors on and between cells. Science. 2024;385(6706):eadl5763. 39024454 10.1126/science.adl5763 PMC12517702

[42] Wang H, Zhang Y, Zeng K, Qiang J, Cao Y, Li Y, et al. Selective mitochondrial protein labeling enabled by biocompatible photocatalytic reactions inside live cells. JACS Au. 2021;1(7):1066–75. 34467350 10.1021/jacsau.1c00172 PMC8395695

[43] Li L, Liang J, Luo H, Tam KM, Tse ECM, Li Y. A new chemical approach for proximity labelling of chromatin-associated RNAs and proteins with visible light irradiation. Chem Commun (Camb). 2019;55(82):12340–3. 31556887 10.1039/c9cc06251c

[44] Geri JB, Oakley JV, Reyes-Robles T, Wang T, McCarver SJ, White CH, et al. Microenvironment mapping via dexter energy transfer on immune cells. Science. 2020;367(6482):1091–7. 32139536 10.1126/science.aay4106 PMC7336666

[45] Seath CP, Burton AJ, Sun X, Lee G, Kleiner RE, MacMillan DWC, et al. Tracking chromatin state changes using nanoscale photo-proximity labelling. Nature. 2023;616(7957):574–80. 37020029 10.1038/s41586-023-05914-y PMC10408239

[46] Oslund RC, Reyes-Robles T, White CH, Tomlinson JH, Crotty KA, Bowman EP, et al. Detection of cell-cell interactions via photocatalytic cell tagging. Nat Chem Biol. 2022;18(8):850–8. 35654846 10.1038/s41589-022-01044-0

[47] Fang Y, Zou P. Genetically encoded photocatalysis for spatiotemporally resolved mapping of biomolecules in living cells and animals. Acc Chem Res. 2025;58(15):2526–34. 40690737 10.1021/acs.accounts.5c00390

[48] Takano T, Wallace JT, Baldwin KT, Purkey AM, Uezu A, Courtland JL, et al. Chemico-genetic discovery of astrocytic control of inhibition in vivo. Nature. 2020;588(7837):296–302. 33177716 10.1038/s41586-020-2926-0 PMC8011649

[49] Kang MG, Rhee HW. Molecular spatiomics by proximity labeling. Acc Chem Res. 2022;55(10):1411–22. 35512328 10.1021/acs.accounts.2c00061 PMC9118551

[50] Chen X, He R, Xiong H, Wang R, Yin Y, Chen Y, et al. Quantitative profiling of lipid transport between organelles enabled by subcellular photocatalytic labelling. Nat Chem. 2025;17(10):1534–45. 40770077 10.1038/s41557-025-01886-w

[51] Kim HB, Kim KE. Precision proteomics with TurboID: mapping the suborganelle landscape. Korean J Physiol Pharmacol. 2024;28(6):495–501. 39467713 10.4196/kjpp.2024.28.6.495 PMC11519719

[52] Bosch JA, Chen CL, Perrimon N. Proximity-dependent labeling methods for proteomic profiling in living cells: an update. Wiley Interdiscip Rev Dev Biol. 2021;10(1):e392. 32909689 10.1002/wdev.392 PMC8142282

[53] Zhou J, Li X, Wen Q, Gan H. Antibody-mediated protein A-APEX2 labeling (AMAPEX) for proximity proteome exploration. Methods Mol Biol. 2025;2953:295–310. 40638056 10.1007/978-1-0716-4694-6_19

[54] Li X, Zhou J, Zhao W, Wen Q, Wang W, Peng H, et al. Defining proximity proteome of histone modifications by antibody-mediated protein A-APEX2 labeling. Genomics Proteomics Bioinform. 2022;20(1):87–100. 10.1016/j.gpb.2021.09.003 PMC9510856 34555496

[55] Duan G, Walther D. The roles of post-translational modifications in the context of protein interaction networks. PLoS Comput Biol. 2015;11(2):e1004049. 25692714 10.1371/journal.pcbi.1004049 PMC4333291

[56] Liu Y, Zeng R, Wang R, Weng Y, Wang R, Zou P, et al. Spatiotemporally resolved subcellular phosphoproteomics. Proc Natl Acad Sci U S A. 2021;118(25):e2025299118. 34135121 10.1073/pnas.2025299118 PMC8237658

[57] Niinae T, Imami K, Sugiyama N, Ishihama Y. Identification of endogenous kinase substrates by proximity labeling combined with kinase perturbation and phosphorylation motifs. Mol Cell Proteomics. 2021;20:100119. 34186244 10.1016/j.mcpro.2021.100119 PMC8325102

[58] Chang L, Chen YJ, Fan CY, Tang CJ, Chen YH, Low PY, et al. Identification of Siglec ligands using a proximity labeling method. J Proteome Res. 2017;16(10):3929–41. 28899088 10.1021/acs.jproteome.7b00625

[59] Liu Y, Nelson ZM, Reda A, Fehl C. Spatiotemporal proximity labeling tools to track GlcNAc sugar-modified functional protein hubs during cellular signaling. ACS Chem Biol. 2022;17(8):2153–64. 35819414 10.1021/acschembio.2c00282 PMC9391317

[60] Xie Y, Sheng Y, Li Q, Ju S, Reyes J, Lebrilla CB. Determination of the glycoprotein specificity of lectins on cell membranes through oxidative proteomics. Chem Sci. 2020;11(35):9501–12. 34094216 10.1039/d0sc04199h PMC8162070

[61] Zhang Z, Wang Y, Lu W, Wang X, Guo H, Pan X, et al. Spatiotemporally resolved mapping of extracellular proteomes via in vivo-compatible TyroID. Nat Commun. 2025;16(1):2553. 40089463 10.1038/s41467-025-57767-w PMC11910615

[62] Kim JW, Yong AJH, Aisenberg EE, Lobel JH, Wang W, Dawson TM, et al. Molecular recording of calcium signals via calcium-dependent proximity labeling. Nat Chem Biol. 2024;20(7):894–905. 38658655 10.1038/s41589-024-01603-7 PMC13296889

[63] Sharma N, Jung M, Mishra PK, Mun JY, Rhee HW. FLEX: genetically encodable enzymatic fluorescence signal amplification using engineered peroxidase. Cell Chem Biol. 2024:S2451-9456(24)00081-3. 38513646 10.1016/j.chembiol.2024.02.007

[64] Kumar S, Javed R, Mudd M, Pallikkuth S, Lidke KA, Jain A, et al. Mammalian hybrid pre-autophagosomal structure HyPAS generates autophagosomes. Cell. 2021;184(24):5950-5969.e22. 34741801 10.1016/j.cell.2021.10.017 PMC8616855

[65] Nalbach K, Schifferer M, Bhattacharya D, Ho-Xuan H, Tseng WC, Williams LA, et al. Spatial proteomics reveals secretory pathway disturbances caused by neuropathy-associated TECPR2. Nat Commun. 2023;14(1):870. 36797266 10.1038/s41467-023-36553-6 PMC9935918

[66] Kim H, Kim KE, Madan E, Martin P, Gogna R, Rhee HW, et al. Unveiling contact-mediated cellular crosstalk. Trends Genet. 2024;40(10):868–79. 38906738 10.1016/j.tig.2024.05.010

[67] Zhang X, Tang Q, Sun J, Guo Y, Zhang S, Liang S, et al. Cellular-scale proximity labeling for recording cell spatial organization in mouse tissues. Sci Adv. 2023;9(21):eadg6388. 37235653 10.1126/sciadv.adg6388 PMC10219591

[68] Pasqual G, Chudnovskiy A, Tas JMJ, Agudelo M, Schweitzer LD, Cui A, et al. Monitoring T cell-dendritic cell interactions in vivo by intercellular enzymatic labelling. Nature. 2018;553(7689):496–500. 29342141 10.1038/nature25442 PMC5853129

[69] Qin W, Cheah JS, Xu C, Messing J, Freibaum BD, Boeynaems S, et al. Dynamic mapping of proteome trafficking within and between living cells by TransitID. Cell. 2023;186(15):3307-3324.e30. 37385249 10.1016/j.cell.2023.05.044 PMC10527209

[70] Cho Y, Jeong I, Kim KE, Rhee HW. Painting cell-cell interactions by horseradish peroxidase and endogenously generated hydrogen peroxide. ACS Chem Biol. 2025;20(1):86–93. 39692451 10.1021/acschembio.4c00419 PMC13459412

[71] Qiu S, Zhao Z, Wu M, Xue Q, Yang Y, Ouyang S, et al. Use of intercellular proximity labeling to quantify and decipher cell-cell interactions directed by diversified molecular pairs. Sci Adv. 2022;8(51):eadd2337. 36542702 10.1126/sciadv.add2337 PMC9770995

[72] Zhang S, Zhao H, Liu Z, Liu K, Zhu H, Pu W, et al. Monitoring of cell-cell communication and contact history in mammals. Science. 2022;378(6623):eabo5503. 36454848 10.1126/science.abo5503

[73] Gräwe C, Stelloo S, van Hout FAH, Vermeulen M. RNA-centric methods: toward the interactome of specific RNA transcripts. Trends Biotechnol. 2021;9(9):890–900. 10.1016/j.tibtech.2020.11.011 33353763

[74] Flynn RA, Pedram K, Malaker SA, Batista PJ, Smith BAH, Johnson AG, et al. Small RNAs are modified with N-glycans and displayed on the surface of living cells. Cell. 2021;184(12):3109-3124.e22. 34004145 10.1016/j.cell.2021.04.023 PMC9097497

[75] McMahon AC, Rahman R, Jin H, Shen JL, Fieldsend A, Luo W, et al. TRIBE: Hijacking an RNA-editing enzyme to identify cell-specific targets of RNA-binding proteins. Cell. 2016;165(3):742–53. 27040499 10.1016/j.cell.2016.03.007 PMC5027142

[76] Kaewsapsak P, Shechner DM, Mallard W, Rinn JL, Ting AY. Live-cell mapping of organelle-associated RNAs via proximity biotinylation combined with protein-RNA crosslinking. Elife. 2017;6:e29224. 29239719 10.7554/eLife.29224 PMC5730372

[77] Benhalevy D, Anastasakis DG, Hafner M. Proximity-CLIP provides a snapshot of protein-occupied RNA elements in subcellular compartments. Nat Methods. 2018;15(12):1074–82. 30478324 10.1038/s41592-018-0220-y PMC6289640

[78] Mukherjee J, Hermesh O, Eliscovich C, Nalpas N, Franz-Wachtel M, Maček B, et al. β-actin mRNA interactome mapping by proximity biotinylation. Proc Natl Acad Sci U S A. 2019;116(26):12863–72. 31189591 10.1073/pnas.1820737116 PMC6600913

[79] Mo J, Chen Z, Cui M, Fang X, Li R, Qin S, et al. In situ proximity labeling of proteins associated with circRNA and RNA G-quadruplexes. Nat Chem Biol. 2025. 10.1038/s41589-025-01993-2 40775047

[80] Li Y, Liu S, Cao L, et al. CBRPP: a new RNA-centric method to study RNA-protein interactions. RNA Biol. 2021;18(11):1608–21. 33596778 10.1080/15476286.2021.1873620 PMC8594927

[81] Fazal FM, Han S, Parker KR, Kaewsapsak P, Xu J, Boettiger AN, et al. Atlas of subcellular RNA localization revealed by APEX-Seq. Cell. 2019;178(2):473-490.e26. 31230715 10.1016/j.cell.2019.05.027 PMC6786773

[82] Padrón A, Iwasaki S, Ingolia NT. Proximity RNA labeling by APEX-Seq reveals the organization of translation initiation complexes and repressive RNA granules. Mol Cell. 2019;75(4):875-887e875. 31442426 10.1016/j.molcel.2019.07.030 PMC6834362

[83] Wang P, Tang W, Li Z, Zou Z, Zhou Y, Li R, et al. Mapping spatial transcriptome with light-activated proximity-dependent RNA labeling. Nat Chem Biol. 2019;15(11):1110–9. 31591565 10.1038/s41589-019-0368-5

[84] Han S, Zhao BS, Myers SA, Carr SA, He C, Ting AY. RNA-protein interaction mapping via MS2- or Cas13-based APEX targeting. Proc Natl Acad Sci U S A. 2020;117(36):22068–79. 32839320 10.1073/pnas.2006617117 PMC7486720

[85] Zhang Z, Sun W, Shi T, Lu P, Zhuang M, Liu JL. Capturing RNA-protein interaction via CRUIS. Nucleic Acids Res. 2020;48(9):e52. 32140725 10.1093/nar/gkaa143 PMC7229851

[86] Lu Z, Gong J, Zhang QC. PARIS: psoralen analysis of RNA interactions and structures with high throughput and resolution. Methods Mol Biol. 2018;1649:59–84. 29130190 10.1007/978-1-4939-7213-5_4 PMC5821472

[87] Myers SA, Wright J, Peckner R, Kalish BT, Zhang F, Carr SA. Discovery of proteins associated with a predefined genomic locus via dCas9-APEX-mediated proximity labeling. Nat Methods. 2018;15(6):437–9. 29735997 10.1038/s41592-018-0007-1 PMC6202184

[88] Gao XD, Tu LC, Mir A, Rodriguez T, Ding Y, Leszyk J, et al. C-BERST: defining subnuclear proteomic landscapes at genomic elements with dCas9-APEX2. Nat Methods. 2018;15(6):433–6. 29735996 10.1038/s41592-018-0006-2 PMC6202229

[89] Qiu W, Xu Z, Zhang M, Zhang D, Fan H, Li T, et al. Determination of local chromatin interactions using a combined CRISPR and peroxidase APEX2 system. Nucleic Acids Res. 2019;47(9):e52. 30805613 10.1093/nar/gkz134 PMC6511869

[90] Liu X, Zhang Y, Chen Y, et al. In situ capture of chromatin interactions by biotinylated dCas9. Cell. 2017;170(5):1028-1043.e19. 28841410 10.1016/j.cell.2017.08.003 PMC6857456

[91] Villaseñor R, Pfaendler R, Ambrosi C, Li M, Zhou F, Li K, et al. ChromID identifies the protein interactome at chromatin marks. Nat Biotechnol. 2020;38(6):728–36. 32123383 10.1038/s41587-020-0434-2 PMC7289633

[92] Qian W, Jiang P, Niu M, Fu Y, Huang D, Zhang D, et al. Selective identification of epigenetic regulators at methylated genomic sites by SelectID. Nat Commun. 2025;16(1):3709. 40251151 10.1038/s41467-025-59002-y PMC12008204

[93] Ding T, Zhu L, Fang Y, Liu Y, Tang W, Zou P. Chromophore-assisted proximity labeling of DNA reveals chromosomal organization in living cells. Angew Chem Int Ed Engl. 2020;59(51):22933–7. 32893421 10.1002/anie.202005486

[94] Kurihara M, Kato K, Sanbo C, Shigenobu S, Ohkawa Y, Fuchigami T, et al. Genomic profiling by ALaP-seq reveals transcriptional regulation by PML bodies through DNMT3A exclusion. Mol Cell. 2020;78(3):493-505.e8. 32353257 10.1016/j.molcel.2020.04.004

[95] Wu F, Olson BG, Yao J. DamID-seq: genome-wide mapping of protein-DNA interactions by high throughput sequencing of adenine-methylated DNA fragments. J Vis Exp. 2016;107:e53620. 10.3791/53620 PMC4781701 26862720

[96] Hanswillemenke A, Hofacker DT, Sorgenfrei M, Fruhner C, Franz-Wachtel M, Schwarzer D, et al. Profiling the interactome of oligonucleotide drugs by proximity biotinylation. Nat Chem Biol. 2024;20(5):555–65. 38233583 10.1038/s41589-023-01530-z PMC11062921

[97] Kwak C, Park C, Ko M, Im CY, Moon H, Park YH, et al. Identification of proteomic landscape of drug-binding proteins in live cells by proximity-dependent target ID. Cell Chem Biol. 2022;29(12):1739-1753.e6. 36272407 10.1016/j.chembiol.2022.10.001

[98] Tao AJ, Jiang J, Gadbois GE, Goyal P, Boyle BT, Mumby EJ, et al. A biotin targeting chimera (BioTAC) system to map small molecule interactomes in situ. Nat Commun. 2023;14(1):8016. 38049406 10.1038/s41467-023-43507-5 PMC10695998

[99] Sun Y, Li C, Deng X, Li W, Deng X, Ge W, et al. Target protein identification in live cells and organisms with a non-diffusive proximity tagging system. Elife. 2024;13: RP102667. 10.7554/eLife.102667 PMC11677243 39728918

[100] Daigle TL, Madisen L, Hage TA, Valley MT, Knoblich U, Larsen RS, et al. A suite of transgenic driver and reporter mouse lines with enhanced brain-cell-type targeting and functionality. Cell. 2018;174(2):465-480.e22. 30007418 10.1016/j.cell.2018.06.035 PMC6086366

[101] MacDonald ML, Ciccimaro E, Prakash A, Banerjee A, Seeholzer SH, Blair IA, et al. Biochemical fractionation and stable isotope dilutionliquid chromatography-mass spectrometry for targeted and microdomain-specific protein quantification in human postmortem brain tissue. Mol Cell Proteomics. 2012;11(12):1670–81. 22942359 10.1074/mcp.M112.021766 PMC3518127

[102] MacDonald ML, Favo D, Garver M, Sun Z, Arion D, Ding Y, et al. Laser capture microdissection-targeted mass spectrometry: a method for multiplexed protein quantification within individual layers of the cerebral cortex. Neuropsychopharmacology. 2019;44(4):743–8. 30390066 10.1038/s41386-018-0260-0 PMC6372704

[103] Mansuri MS, Peng G, Wilson RS, Lam TT, Zhao H, Williams KR, et al. Differential protein expression in striatal D1-and D2-dopamine receptor-expressing medium spiny neurons. Proteomes. 2020;8(4):27. 33066078 10.3390/proteomes8040027 PMC7709116

[104] Sun M, Yuan F, Tang Y, Zou P, Lei X. Subcellular interactomes revealed by merging APEX with cross-linking mass spectrometry. Anal Chem. 2022;94(43):14878–88. 36265550 10.1021/acs.analchem.2c02116

[105] Wang H, Wang Z, Gao H, Liu J, Qiao Z, Zhao B, et al. A photo-oxidation driven proximity labeling strategy enables profiling of mitochondrial proteome dynamics in living cells. Chem Sci. 2022;13(40):11943–50. 36320915 10.1039/d2sc04087e PMC9580500

[106] Liu Z, Guo F, Zhu Y, Qin S, Hou Y, Guo H, et al. Bioorthogonal photocatalytic proximity labeling in primary living samples. Nat Commun. 2024;15(1):2712. 38548729 10.1038/s41467-024-46985-3 PMC10978841

[107] Park I, Kim KE, Kim J, Kim AK, Bae S, Jung M, et al. Mitochondrial matrix RTN4IP1/OPA10 is an oxidoreductase for coenzyme Q synthesis. Nat Chem Biol. 2024;20(2):221–33. 37884807 10.1038/s41589-023-01452-w PMC10830421

[108] Cho KF, Branon TC, Rajeev S, Svinkina T, Udeshi ND, Thoudam T, et al. Split-TurboID enables contact-dependent proximity labeling in cells. Proc Natl Acad Sci U S A. 2020;117(22):12143–54. 32424107 10.1073/pnas.1919528117 PMC7275672

[109] Hung V, Lam SS, Udeshi ND, Svinkina T, Guzman G, Mootha VK, et al. Proteomic mapping of cytosol-facing outer mitochondrial and ER membranes in living human cells by proximity biotinylation. Elife. 2017;6:e24463. 28441135 10.7554/eLife.24463 PMC5404927

[110] Xu F, Du W, Zou Q, Wang Y, Zhang X, Xing X, et al. COPII mitigates ER stress by promoting formation of ER whorls. Cell Res. 2021;31(2):141–56. 32989223 10.1038/s41422-020-00416-2 PMC8026990

[111] Lyu Z, Sycks MM, Espinoza MF, Nguyen KK, Montoya MR, Galapate CM, et al. Monitoring protein import into the endoplasmic reticulum in living cells with proximity labeling. ACS Chem Biol. 2022;17(7):1963–77. 35675579 10.1021/acschembio.2c00405

[112] Kim KE, Park I, Kim J, Kang MG, Choi WG, Shin H, et al. Dynamic tracking and identification of tissue-specific secretory proteins in the circulation of live mice. Nat Commun. 2021;12(1):5204. 34471136 10.1038/s41467-021-25546-y PMC8410947

[113] van Leeuwen W, VanInsberghe M, Battich N, Salmén F, van Oudenaarden A, Rabouille C. Identification of the stress granule transcriptome via RNA-editing in single cells and in vivo. Cell Rep Methods. 2022;2(6):10023. 10.1016/j.crmeth.2022.100235 PMC9243631 35784648

[114] Ren Z, Li R, Zhou X, Chen Y, Fang Y, Zou P. Enzyme-mediated proximity labeling identifies small RNAs in the endoplasmic reticulum lumen. Biochemistry. 2023;62(12):1844–8. 37253270 10.1021/acs.biochem.3c00142

[115] Zhang Y, Liu Z, Zhou N, Guo F, Guo H, Chen X, et al. In situ lysosomal proteomics enabled by bioorthogonal photocatalytic proximity labelling. Nat Catal. 2025;8:162–77.

[116] Frankenfield AM, Fernandopulle MS, Hasan S, Ward ME, Hao L. Development and comparative evaluation of endolysosomal proximity labeling-cased proteomic methods in human iPSC-derived neurons. Anal Chem. 2020;92(23):15437–44. 33201688 10.1021/acs.analchem.0c03107 PMC8895409

[117] Tan JX, Finkel T. A phosphoinositide signalling pathway mediates rapid lysosomal repair. Nature. 2022;609(7928):815–21. 36071159 10.1038/s41586-022-05164-4 PMC9450835

[118] Bhattacharya A, Mukherjee R, Kuncha SK, Brunstein ME, Rathore R, Junek S, et al. A lysosome membrane regeneration pathway depends on TBC1D15 and autophagic lysosomal reformation proteins. Nat Cell Biol. 2023;25(5):685–98. 37024685 10.1038/s41556-023-01125-9

[119] Frankenfield A, Ni J, Hao L. Characterization of neuronal lysosome interactome with proximity labeling proteomics. J Vis Exp. 2022:(184):10.3791/64132. 10.3791/64132 PMC9997703 35815987

[120] Özkan N, Koppers M, van Soest I, van Harten A, Jurriens D, Liv N, et al. ER - lysosome contacts at a pre-axonal region regulate axonal lysosome availability. Nat Commun. 2021;12(1):4493. 34301956 10.1038/s41467-021-24713-5 PMC8302662

[121] Ren Z, Tang W, Peng L, Zou P. Profiling stress-triggered RNA condensation with photocatalytic proximity labeling. Nat Commun. 2023;14(1):7390. 37968266 10.1038/s41467-023-43194-2 PMC10651888

[122] Markmiller S, Soltanieh S, Server KL, Mak R, Jin W, Fang MY, et al. Context-dependent and disease-specific diversity in protein interactions within stress granules. Cell. 2018;172(3):590-604.e13. 29373831 10.1016/j.cell.2017.12.032 PMC5969999

[123] Hamdan H, Lim BC, Torii T, Joshi A, Konning M, Smith C, et al. Mapping axon initial segment structure and function by multiplexed proximity biotinylation. Nat Commun. 2020;11(1):100. 31900387 10.1038/s41467-019-13658-5 PMC6941957

[124] Zhang W, Fu Y, Peng L, Ogawa Y, Ding X, Rasband A, et al. Immunoproximity biotinylation reveals the axon initial segment proteome. Nat Commun. 2023;14(1):8201. 38081810 10.1038/s41467-023-44015-2 PMC10713531

[125] Falahati H, Wu Y, Feuerer V, Simon HG, De Camilli P. Proximity proteomics of synaptopodin provides insight into the molecular composition of the spine apparatus of dendritic spines. Proc Natl Acad Sci U S A. 2022;119(42):e2203750119. 36215465 10.1073/pnas.2203750119 PMC9586327

[126] Smirnova EV, Rakitina TV, Ziganshin RH, Saratov GA, Arapidi GP, Belogurov AA Jr, et al. Identification of myelin basic protein proximity interactome using TurboID labeling proteomics. Cells. 2023;12(6):944. 36980286 10.3390/cells12060944 PMC10047773

[127] Takato M, Sakamoto S, Nonaka H, Tanimura Valor FY, Tamura T, Hamachi I. Photoproximity labeling of endogenous receptors in the live mouse brain in minutes. Nat Chem Biol. 2025;21(1):109–19. 39090312 10.1038/s41589-024-01692-4

[128] Dumrongprechachan V, Salisbury RB, Soto G, Kumar M, MacDonald ML, Kozorovitskiy Y. Cell-type and subcellular compartment-specific APEX2 proximity labeling reveals activity-dependent nuclear proteome dynamics in the striatum. Nat Commun. 2021;12(1):4855. 34381044 10.1038/s41467-021-25144-y PMC8357913

[129] Dumrongprechachan V, Salisbury RB, Butler L, MacDonald ML, Kozorovitskiy Y. Dynamic proteomic and phosphoproteomic atlas of corticostriatal axons in neurodevelopment. Elife. 2022;11:e78847. 36239373 10.7554/eLife.78847 PMC9629834

[130] Bartolomé-Martín D, Ibáñez I, Piniella D, Martínez-Blanco E, Pelaz SG, Zafra F. Identification of potassium channel proteins Kv7.2/7.3 as common partners of the dopamine and glutamate transporters DAT and GLT-1. Neuropharmacology. 2019;161:107568. 30885609 10.1016/j.neuropharm.2019.03.011

[131] Sun X, Sun H, Han X, Chen PC, Jiao Y, Wu Z, et al. Deep single-cell-type proteome profiling of mouse brain by nonsurgical AAV-mediated proximity labeling. Anal Chem. 2022;94(13):5325–34. 35315655 10.1021/acs.analchem.1c05212 PMC9350993

[132] Irala D, Wang S, Sakers K, Nagendren L, Ulloa-Severino FP, Bindu DS, et al. Astrocyte-secreted neurocan controls inhibitory synapse formation and function. Neuron. 2024;112(10):1657-1675.e10. 38574730 10.1016/j.neuron.2024.03.007 PMC11098688

[133] Tetenborg S, Shihabeddin E, Kumar EOAM, Sigulinsky C, Dedek K, Lin YP, et al. Uncovering the electrical synapse proteome in retinal neurons via in vivo proximity labeling. bioRxiv [Preprint]. 2025. 10.1101/2024.11.26.625481. 10.7554/eLife.105935 PMC13189625 42159345

[134] Xie S, Li H, Yao F, Huang J, Yang X, Chen X, et al. PUPIL enables mapping and stamping of transient electrical connectivity in developing nervous systems. Cell Rep. 2021;37(3):109853. 34686323 10.1016/j.celrep.2021.109853

[135] Killinger BA, Mercado G, Choi S, Tittle T, Chu Y, Brundin P, et al. Distribution of phosphorylated alpha-synuclein in non-diseased brain implicates olfactory bulb mitral cells in synucleinopathy pathogenesis. NPJ Parkinsons Dis. 2023;9(1):43. 36966145 10.1038/s41531-023-00491-3 PMC10039879

[136] Zhu H, Oh JH, Matsuda Y, Mino T, Ishikawa M, Nakamura H, et al. Tyrosinase-based proximity labeling in living cells and in vivo. J Am Chem Soc. 2024;146(11):7515–23. 38445591 10.1021/jacs.3c13183

[137] Bowen CA, Nguyen HM, Lin Y, Bagchi P, Natu A, Espinosa-Garcia C, et al. Proximity labeling proteomics reveals Kv1.3 potassium channel immune interactors in microglia. Mol Cell Proteomics. 2024;23(8):100809. 38936775 10.1016/j.mcpro.2024.100809 PMC11780389

[138] Allendorf DH, Franssen EH, Brown GC. Lipopolysaccharide activates microglia via neuraminidase 1 desialylation of Toll-like receptor 4. J Neurochem. 2020;155(4):403–16. 32279315 10.1111/jnc.15024

[139] Sunna S, Bowen C, Zeng H, Rayaprolu S, Kumar P, Bagchi P, et al. Cellular proteomic profiling using proximity labeling by TurboID-NES in microglial and neuronal cell lines. Mol Cell Proteomics. 2023;22(6):100546. 37061046 10.1016/j.mcpro.2023.100546 PMC10205547

[140] Cijsouw T, Ramsey AM, Lam TT, Carbone BE, Blanpied TA, Biederer T. Mapping the proteome of the synaptic cleft through proximity labeling reveals new cleft proteins. Proteomes. 2018;6(4):48. 30487426 10.3390/proteomes6040048 PMC6313906

[141] Rayaprolu S, Bitarafan S, Santiago JV, Betarbet R, Sunna S, Cheng L, et al. Cell type-specific biotin labeling in vivo resolves regional neuronal and astrocyte proteomic differences in mouse brain. Nat Commun. 2022;13(1):2927. 35614064 10.1038/s41467-022-30623-x PMC9132937

[142] Soto JS, Jami-Alahmadi Y, Wohlschlegel JA, Khakh BS. In vivo identification of astrocyte and neuron subproteomes by proximity-dependent biotinylation. Nat Protoc. 2024;19(3):896–927. 38062165 10.1038/s41596-023-00923-7 PMC11917372

[143] Cai Y, Pinheiro-de-Sousa I, Slobodyanyuk M, Chen F, Huynh T, Kanyo J, et al. Myelin-axon interface vulnerability in Alzheimer’s disease revealed by subcellular proteomics and imaging of human and mouse brain. Nat Neurosci. 2025;28(7):1418–35. 40514588 10.1038/s41593-025-01973-8 PMC12395420

[144] Shapley SM, Shantaraman A, Gadhavi J, Kearney MA, Dammer EB, Duong DM, et al. Proximity labeling of the Tau repeat domain enriches RNA-binding proteins that are altered in Alzheimer’s disease and related tauopathies. Mol Cell Proteomics. 2025;25(1):101458. 41205924 10.1016/j.mcpro.2025.101458 PMC12796112

[145] Tracy TE, Madero-Pérez J, Swaney DL, Chang TS, Moritz M, Konrad C, et al. Tau interactome maps synaptic and mitochondrial processes associated with neurodegeneration. Cell. 2022;185(4):712-728.e14. 35063084 10.1016/j.cell.2021.12.041 PMC8857049

[146] Prikas E, Paric E, Asih PR, Stefanoska K, Stefen H, Fath T, et al. Tau target identification reveals NSF-dependent effects on AMPA receptor trafficking and memory formation. EMBO J. 2022;41(18):e10242. 35993331 10.15252/embj.2021110242 PMC9475529

[147] Yu H, Liu D, Zhang Y, Tang R, Tang R, Fan X, et al. Tissue-specific O-GlcNAcylation profiling identifies substrates in translational machinery in Drosophila mushroom body contributing to olfactory learning. Elife. 2024;13:e91269. 38619103 10.7554/eLife.91269 PMC11018347

[148] Cai Y, Kanyo J, Wilson R, Bathla S, Cardozo PL, Tong L, et al. Subcellular proteomics and iPSC modeling uncover reversible mechanisms of axonal pathology in Alzheimer’s disease. Nat Aging. 2025;5(3):504–27. 40065072 10.1038/s43587-025-00823-3 PMC11922768

[149] Gabriel JL, Tinti M, Fuller W, Ashford MLJ. Identifying the beta-site amyloid precursor protein cleaving enzyme 1 interactome through the proximity-dependent biotin identification assay. Neurosci Lett. 2022;767:136302. 34710551 10.1016/j.neulet.2021.136302

[150] Chung CY, Khurana V, Yi S, Sahni N, Loh KH, Auluck PK, et al. In situ peroxidase labeling and mass-spectrometry connects alpha-synuclein directly to endocytic trafficking and mRNA metabolism in neurons. Cell Syst. 2017;4(2):242-250.e4. 28131823 10.1016/j.cels.2017.01.002 PMC5578869

[151] Lee JY, Kim H, Jo A, Khang R, Park CH, Park SJ, et al. α-Synuclein A53T binds to transcriptional adapter 2-alpha and blocks histone H3 acetylation. Int J Mol Sci. 2021;22(10):5392. 34065515 10.3390/ijms22105392 PMC8161267

[152] Killinger BA, Marshall LL, Chatterjee D, Chu Y, Bras J, Guerreiro R, et al. In situ proximity labeling identifies Lewy pathology molecular interactions in the human brain. Proc Natl Acad Sci U S A. 2022;119(5):e2114405119. 35082147 10.1073/pnas.2114405119 PMC8812572

[153] Zhai Y, Huang X, Zhang K, Huang Y, Jiang Y, Cui J, et al. Spatiotemporal-resolved protein networks profiling with photoactivation dependent proximity labeling. Nat Commun. 2022;13(1):4906. 35987950 10.1038/s41467-022-32689-z PMC9392063

[154] Johnson MA, Nuckols TA, Merino P, Bagchi P, Nandy S, Root J, et al. Proximity-based labeling reveals DNA damage-induced phosphorylation of fused in sarcoma (FUS) causes distinct changes in the FUS protein interactome. J Biol Chem. 2022;298(8):102135. 35709984 10.1016/j.jbc.2022.102135 PMC9372748

[155] Wei S, Yang Y, Wang Y. Proximity proteomics revealed aberrant mRNA splicing elicited by ALS-linked profilin-1 mutants. Anal Chem. 2023;95(41):15141–5. 37787459 10.1021/acs.analchem.3c03734 PMC10689300

[156] Chen X, He X, Yang YY, Wang Y. Amyotrophic lateral sclerosis-associated mutants of SOD1 modulate miRNA biogenesis through aberrant interactions with Exportin 5. ACS Chem Biol. 2022;17(12):3450–7. 36475596 10.1021/acschembio.2c00591 PMC9867941

[157] Xie L, Zhu Y, Hurtle BT, Wright M, Robinson JL, Mauna JC, et al. Context-dependent interactors regulate TDP-43 dysfunction in ALS/FTLD. bioRxiv [Preprint]. 2025. 10.1101/2025.04.07.646890.

[158] Deng X, Bradshaw G, Kalocsay M, Mitchison T. Tubulin regulates the stability and localization of STMN2 by binding preferentially to its soluble form. bioRxiv. 2025. 10.1101/2025.02.27.640326. 41171096 10.1083/jcb.202502192 PMC12803766

[159] Liao YC, Fernandopulle MS, Wang G, Choi H, Hao L, Drerup CM, et al. RNA granules hitchhike on lysosomes for long-distance transport, using Annexin A11 as a molecular tether. Cell. 2019;179(1):147-164.e20. 31539493 10.1016/j.cell.2019.08.050 PMC6890474

[160] Mosti F, Hoye ML, Escobar-Tomlienovich CF, Silver DL. Multi-modal investigation reveals pathogenic features of diverse DDX3X missense mutations. PLoS Genet. 2025;21(1):e1011555. 39836689 10.1371/journal.pgen.1011555 PMC11771946

[161] Murtaza N, Cheng AA, Brown CO, Meka DP, Hong S, Uy JA, et al. Neuron-specific protein network mapping of autism risk genes identifies shared biological mechanisms and disease-relevant pathologies. Cell Rep. 2022;41(8):111678. 36417873 10.1016/j.celrep.2022.111678

[162] Unda BK, Chalil L, Yoon S, Kilpatrick S, Irwin C, Xing S, et al. Impaired OTUD7A-dependent Ankyrin regulation mediates neuronal dysfunction in mouse and human models of the 15q13. 3 microdeletion syndrome. Mol psychiatry. 2023;28(4):1747–69. 36604605 10.1038/s41380-022-01937-5 PMC10208958

[163] Yan Q, Wulfridge P, Doherty J, Fernandez-Luna JL, Real PJ, Tang HY, et al. Proximity labeling identifies a repertoire of site-specific R-loop modulators. Nat Commun. 2022;13(1):5. 35013239 10.1038/s41467-021-27722-6 PMC8748879

[164] Feng H, Zhao Q, Zhao N, Liang Z, Huang Y, Zhang X, et al. A cell-permeable photosensitizer for selective proximity labeling and crosslinking of aggregated proteome. Adv Sci (Weinh). 2024;11(18):e2306950. 38441365 10.1002/advs.202306950 PMC11095223

[165] Teixeira M, Sheta R, Musiol D, Ranjakasoa V, Loehr J, Lambert JP, et al. Combining light-induced aggregation and biotin proximity labeling implicates endolysosomal proteins in early α-synuclein oligomerization. iScience. 2025;28(7):112823. 40612512 10.1016/j.isci.2025.112823 PMC12221657

[166] Sun R, Huang Y, Feng H, Zhao N, Wan W, Shen D, et al. 1000 fold ultra-photosensitized fluorescent protein mimics toward photocatalytic proximity labeling and proteomic profiling functions. Adv Sci. 2025;12(15):e2413063. 10.1002/advs.202413063 PMC12005797 39985251

[167] Li K, Xie X, Gao R, Chen Z, Yang M, Wen Z, et al. Spatiotemporal protein interactome profiling through condensation-enhanced photocrosslinking. Nat Chem. 2025;17(1):111–23. 39501047 10.1038/s41557-024-01663-1

[168] Božič J, Motaln H, Janež AP, Markič L, Tripathi P, Yamoah A, et al. Interactome screening of C9orf72 dipeptide repeats reveals VCP sequestration and functional impairment by polyGA. Brain. 2022;145(2):684–99. 34534264 10.1093/brain/awab300 PMC9014755

[169] Batra S, Vaquer-Alicea J, Valdez C, Taylor SP, Manon VA, Vega AR, et al. VCP regulates early tau seed amplification via specific cofactors. Mol Neurodegener. 2025;20(1):2. 39773263 10.1186/s13024-024-00783-z PMC11707990

[170] Morderer D, Wren MC, Liu F, Kouri N, Maistrenko A, Khalil B, et al. Probe-dependent proximity profiling (ProPPr) uncovers similarities and differences in phospho-tau-associated proteomes between tauopathies. Mol Neurodegener. 2025;20(1):32. 40082954 10.1186/s13024-025-00817-0 PMC11905455

[171] Choi SG, Tittle TR, Barot RR, Betts DJ, Gallagher JJ, Kordower JH, et al. Proximity proteomics reveals unique and shared pathological features between multiple system atrophy and Parkinson’s disease. Acta Neuropathol Commun. 2025;13(1):65. 40122840 10.1186/s40478-025-01958-5 PMC11931798

[172] Kumar P, Goettemoeller AM, Espinosa-Garcia C, Tobin BR, Tfaily A, Nelson RS, et al. Native-state proteomics of parvalbumin interneurons identifies unique molecular signatures and vulnerabilities to early Alzheimer’s pathology. Nat Commun. 2024;15(1):2823. 38561349 10.1038/s41467-024-47028-7 PMC10985119

[173] Peng Q, Weerapana E. Profiling nuclear cysteine ligandability and effects on nuclear localization using proximity labeling-coupled chemoproteomics. Cell Chem Biol. 2024;31(3):550-564.e9. 38086369 10.1016/j.chembiol.2023.11.010 PMC10960692

[174] Hobson BD, Choi SJ, Mosharov EV, Soni RK, Sulzer D, Sims PA. Subcellular proteomics of dopamine neurons in the mouse brain. Elife. 2022;11:e70921. 35098924 10.7554/eLife.70921 PMC8860448

[175] Hasan S, Fernandopulle MS, Humble SW, Frankenfield AM, Li H, Prestil R, et al. Multi-modal proteomic characterization of lysosomal function and proteostasis in progranulin-deficient neurons. Mol Neurodegener. 2023;18(1):87. 37974165 10.1186/s13024-023-00673-w PMC10655356

[176] Martija AA, Krauß A, Bächle N, Doth L, Christians A, Krunic D, et al. EMP3 sustains oncogenic EGFR/CDK2 signaling by restricting receptor degradation in glioblastoma. Acta Neuropathol Commun. 2023;11(1):177. 37936247 10.1186/s40478-023-01673-z PMC10629159

[177] Tong F, Zhou W, Janiszewska M, Seath CP. Multiprobe photoproximity labeling of the EGFR interactome in glioblastoma using red-light. J Am Chem Soc. 2025;147(11):9316–27. 40052329 10.1021/jacs.4c15537 PMC12532021

[178] Hu M, Weldy A, Lovalvo I, Akins E, Jain S, Chang A, et al. Druggable genome CRISPRi screen in 3D hydrogels reveals regulators of cortactin-driven actin remodeling in invading glioblastoma cells. bioRxiv. 2025. 10.1101/2025.01.20.633978.41573943

[179] Shin S, Lee SY, Kang MG, Jang DG, Kim J, Rhee HW, et al. Super-resolution proximity labeling with enhanced direct identification of biotinylation sites. Commun Biol. 2024;7(1):554. 38724559 10.1038/s42003-024-06112-w PMC11082246

[180] Kim HB, Kim KE. A straightforward interpretation of proximity labeling through direct biotinylation analysis. ACS Omega. 2025;10(24):26098–105. 40584372 10.1021/acsomega.5c03099 PMC12199003

[181] Scipioni L, Tedeschi G, Navarro MX, Jia YY, Zhu S, Halbers LP, et al. ESPRESSO: spatiotemporal omics based on organelle phenotyping. Nat Methods. 2025;22(11):2349–61.41184551 10.1038/s41592-025-02863-4

